# Molecular and cellular insights into the parasitic infection of bloom-forming marine diatoms by *Pirsonia diadema*

**DOI:** 10.1038/s41564-026-02421-4

**Published:** 2026-07-21

**Authors:** Varsha Mathur, Nicholas A. T. Irwin, Luis Javier Galindo, Elisabet Alacid, Kevin Moog, Elliot Flaum, Gautam Dey, Flora Vincent, Michael Cunliffe, Sunju Kim, Ulrich Technau, Thomas A. Richards

**Affiliations:** 1 Department of Biology, University of Oxford, Oxford, UK; 2 Faculty of Life Sciences, University of Vienna, Vienna, Austria; 3 Gregor Mendel Institute (GMI), Austrian Academy of Sciences, Vienna BioCenter (VBC), Vienna, Austria; 4 Institute of Water Research, University of Granada, Granada, Spain; 5 Department of Ecology, University of Granada, Granada, Spain; 6 Centre d’Estudis Avançats de Blanes, Blanes, Spain; 7 Developmental Biology Unit, European Molecular Biology Laboratory, Heidelberg, Germany; 8 Cell Biology and Biophysics Unit, European Molecular Biology Laboratory, Heidelberg, Germany; 9 School of Biological and Marine Sciences, University of Plymouth, Plymouth, UK; 10 Major of Oceanography, Division of Earth Environmental System Science, Pukyong National University, Busan, Republic of Korea

**Keywords:** Symbiosis, Parasite evolution, Parasite genomics, Parasite biology, Microbial ecology

## Abstract

Microbial host–parasite interactions shape global biogeochemical cycles, yet their cellular and evolutionary mechanisms remain poorly understood. Here we developed a model pathosystem to explore the interaction between the eukaryotic microparasite, *Pirsonia diadema*, and its host, the bloom-forming diatom, *Coscinodiscus*. Culture conditions recapitulated *Pirsonia*’s rapid life cycle in vitro, and live-cell imaging enabled the quantitative analysis of infection dynamics and host mortality. Genome sequencing and time-resolved dual RNA sequencing of *Pirsonia* revealed unique genetic innovations relative to their oomycete relatives and an upregulation of an expanded multi-gene family of integrin-like proteins in *Pirsonia* zoospores during infection. Targeted drug perturbations combined with ultrastructure expansion microscopy confirm stage specific roles for *Pirsonia*’s cytoskeleton, including actin-dependent host attachment and formation of intracellular parasite-induced feeding structures and microtubule-associated processes in zoospore differentiation. Together, these findings reveal mechanisms underpinning *Pirsonia* infection and provide insights into the evolution of strategies that underpin parasitic shunts in marine trophic networks.

## Abstract (web summary)

Ultrastructure expansion microscopy, live-cell imaging and time-resolved dual RNA sequencing reveal the infection dynamics of a microparasite, providing insights into how *Pirsonia diadema* shapes marine trophic networks.

## Main

The ocean contains diverse microorganisms, including bacteria, archaea, viruses and microbial eukaryotes^1,2^. These species form the base of marine trophic webs and drive the biogeochemical cycling of vital nutrients, particularly fixed carbon and nitrogen^3^. Eukaryotic phytoplankton, such as diatoms and coccolithophores, account for 1–2% of Earth’s primary producer biomass yet are responsible for 40% of global annual carbon-based photosynthesis^4^. This is partly driven by algal blooms, which form through rapid proliferation^5^. These blooms have manifold ecological implications: they can produce harmful toxins^6^ but also form hotspots for microbial interactions with large-scale impacts on marine metabolic flux and, ultimately, global geochemical cycles^7^. Accordingly, understanding the ecological and cellular interactions governing bloom dynamics is important for understanding ocean ecosystems.

Although nutrient availability and climatic conditions can drive bloom formation, grazing by predators and pathogen infections have a major role in bloom collapse^8^. In particular, viral infections impact bloom dynamics and carbon flow through the ‘viral shunt’^9,10^. Similarly, eukaryotic microparasites are important agents of algal mortality, regulating diverse phytoplankton such as dinoflagellates and diatoms^11–13^, and are consistently found at high abundance in metabarcoding surveys of ocean environments^14^. Despite this, the impact of microparasites on planktonic networks and biogeochemistry is rarely considered^2^. Establishing experimentally tractable pathosystems has been challenging owing to the complexities of parasite isolation and host–parasite co-culture maintenance. Nonetheless, a few model pathosystems have shed light on metabolic and transcriptomic reprogramming during parasitic infections^15–17^. In addition, single-cell transcriptomics and metabarcoding have facilitated studies of uncultured marine parasites^18,19^. However, developing diverse, tractable host–parasite model systems remains critical for gaining genomic and cellular insights into these interactions.

To address this, we established a tractable marine model pathosystem involving the globally distributed parasite *Pirsonia diadema* and its diatom hosts, *Coscinodiscus. Pirsonia* is a relative of the Pseudofungi^20^, a group that includes the oomycetes, and exhibits a parasitoid life strategy, exploiting the host for development, ultimately leading to host mortality^21,22^. *P. diadema* is a parasite of the centric diatom genus *Coscinodiscus* and infects *C. granii*, *C. radiatus*, *C. wailesii* and *C. concinnus*^23^*. Coscinodiscus* form seasonal blooms and are protected by a robust silica cell wall, known as the frustule^24^. To understand the molecular and cellular dynamics underpinning this interaction, we established long-term co-cultures of *Pirsonia* and *Coscinodiscus*. Live-cell imaging enabled quantitative assessments of infection progression and genomic and transcriptomic analyses revealed lineage-specific innovations in parasitic surface proteins and cytoskeletal regulators critical for infection. These data illuminate parasite evolution and cellular mechanisms in this key marine microbial interaction.

## Results

### Establishing a diatom-parasite pathosystem

**Fig. 1:**
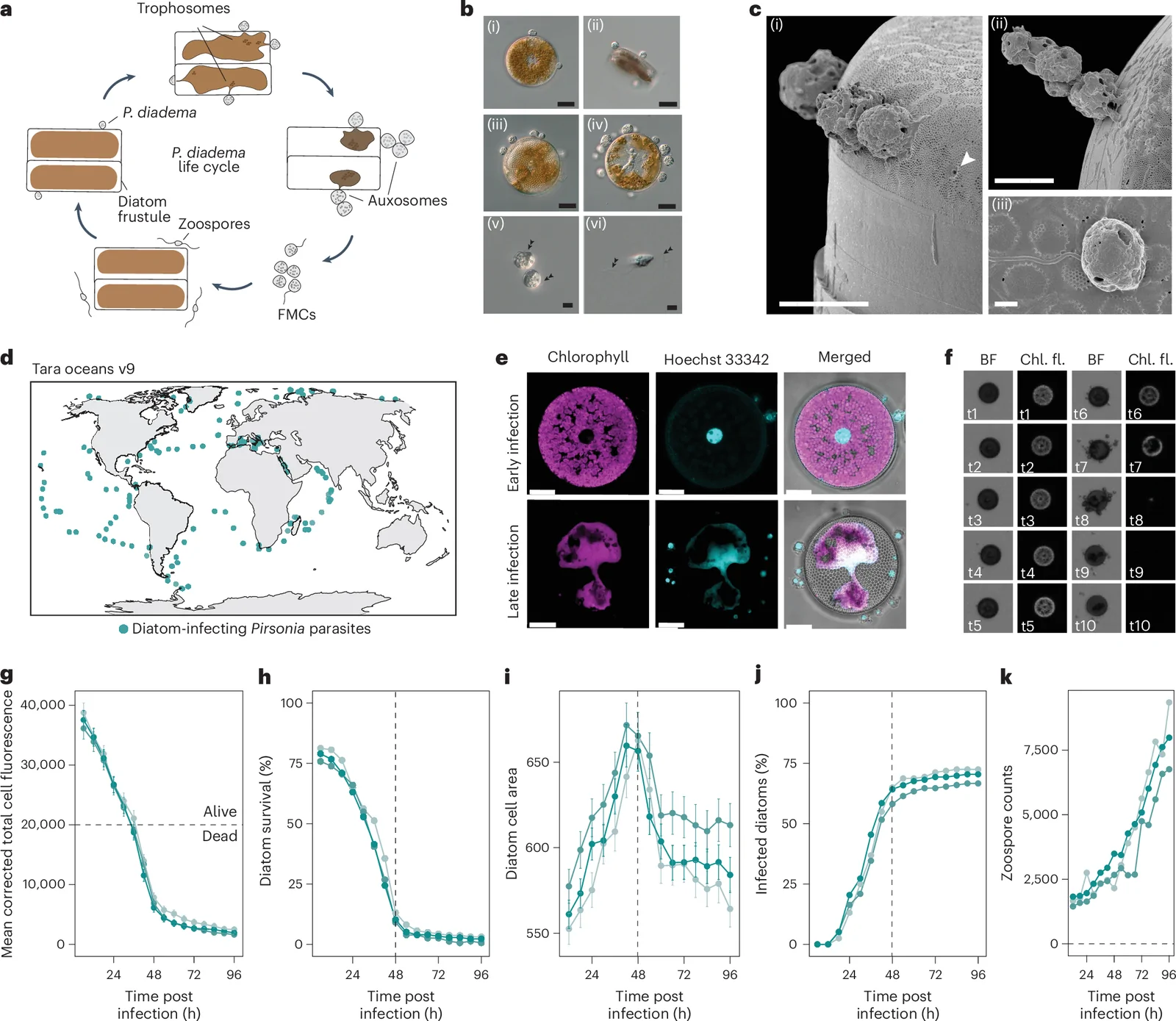
Microscopic characterization of the *Pirsonia*–*Coscinodiscus* model system. **a**, A schematic of the developmental stages and life cycle of *P. diadema* with *Coscinodiscus* shown in girdle view**. b**, Light micrographs of *P. diadema* infecting *C. radiatus* (top) and two flagellate stages: FMCs and zoospores (bottom). Scale bars, 20 μm (i–iv), 5 μm (v and vi). **c**, The frustule surface host–parasite interaction visualized by SEM. White arrow indicates rimoportulae (sites of parasite attachment). Scale bars, 5 μm (i and ii), 1 μm (iii). Images in **b** and **c** are representative of observations from at least three independent infections. **d**, The geographical distribution of 18S rDNA V9 region in environmental OTUs assigned to *Pirsonia* in the Tara Oceans Expedition. **e**, The fluorescence microscopy of early and late infections. Chlorophyll autofluorescence (Chl. fl.)-purple and Hoechst 33342 DNA-light blue staining of *C. radiatus* infected with *P. diadema*. Scale bars, 10 μm. **f**, An automated live-cell high-content screening (HCS) of *P. diadema* infection. Brightfield (BF) and Chl. fl. micrographs show one cell tracked every 6 h over 4 days. t, timepoint. **g**, The mean CTCF of infected diatoms over time. The horizontal dashed line indicates the Chl. fl. threshold for cell viability. **h**, The diatom survival curve calculated on the basis of CTCF levels. The vertical dashed line marks the timepoint where widespread cell death occurred. **i**, The diatom cell area over time post infection. The vertical dashed line marks the timepoint where cell area peaks. **j**, The percentage of infected diatoms over time based on area, plateauing after ~48 h (vertical dashed line). The proportion of infected cells does not reach 100% owing to the presence of several non-viable diatoms at the start of the experiment. **k**, The line graph displaying zoospore numbers over time post infection. In **g**–**k**, the HCS quantification of infection dynamics in *P. diadema*-infected diatom cells (*n* = 489 cells tracked across three wells) is shown. Each line represents one well (biological replicate). In **g** and **i**, points show the mean of tracked cells within each well at each timepoint, with error bars indicating standard error (s.d./√*n*) calculated from cell-level measurements.

To investigate the *Pirsonia*–*Coscinodiscus* interaction, we first established a stable co-culture by performing single-cell isolations of *Coscinodiscus* and generating clonal host cultures from natural plankton samples collected from the Western English Channel, UK. *P. diadema* was isolated by manually picking infected *Coscinodiscus* cells from Yongho Bay in Busan, Republic of Korea^21^. We observed *P. diadema* life cycle and cellular morphology using light microscopy and scanning electron microscopy (SEM) (Fig. 1a–c). In culture, we observed the motile, bi-flagellate zoospores of *Pirsonia* attached to the diatom frustule, specifically at diatom pores, known as rimoportulae^25^ (Fig. 1c, white arrow). The zoospores lose their flagella and differentiate into auxosomes, the main parasitic cell body, many of which are frequently found on the surface of a single host cell (Fig. 1b). Whereas the auxosome remains on the diatom surface, growing and dividing, the diatom protoplast is remodelled into digestive intracellular compartments known as trophosomes^26^. The auxosome and trophosome are connected by a thin cytoplasmic strand through which digested host contents pass. After host death, auxosomes detach from the trophosomes and form flagellate mother cells (FMCs), which possess a single flagellum and are motile, leaving behind an empty diatom frustule. The FMCs eventually divide and differentiate into numerous bi-flagellated, fast-swimming, infective zoospores, completing the previously described parasitoid life cycle^22^. Under laboratory culture conditions, this process invariably resulted in host death, and no diatoms were observed to be resistant to infection.

Having observed the complete pathogenic life cycle in the laboratory, we next assessed the ecological relevance of *Pirsonia* parasites using environmental sequencing data. We analysed the 18S ribosomal DNA (rDNA) V9 region metabarcoding dataset from the Tara Oceans Expedition^27^ and identified operational taxonomic units (OTUs) corresponding to *Pirsonia* species that infect diverse diatoms across global oceans (Fig. 1d and Supplementary Table 1). This broad distribution underscores their ecological significance in global planktonic systems.

### The temporal dynamics of the *Pirsonia*–*Coscinodiscus* infection cycle

To gain deeper insight into cellular changes during infection, we used fluorescence confocal microscopy (Fig. 1e). During early infection, the diatom protoplast remains intact, with the nucleus centred and surrounded by a dense network of chloroplasts exhibiting strong chlorophyll autofluorescence (Fig. 1e, top). However, later in infection, we observed a marked decrease in chlorophyll autofluorescence and DNA staining, as the diatom protoplasm is degraded by *Pirsonia* trophosomes (Fig. 1e, bottom).

Leveraging chlorophyll autofluorescence loss as a proxy for host viability and infection progression, we quantified infection dynamics using an automated live-cell high-content screening (HCS) screener. This approach allowed tracking of individual diatom cells (*n* = 489) following exposure to *Pirsonia* zoospores, with imaging every 6 h over 4 days (Fig. 1f and Supplementary Video 1). *Coscinodiscus* chlorophyll autofluorescence was measured over time and quantified as mean corrected total cell fluorescence (CTCF). CTCF declined within 12 h post infection, and diatoms were classified as non-viable once CTCF dropped below 50% of their initial value (Fig. 1f–h). Using this threshold, survival curves showed that most host cells died within 48 h of infection (Fig. 1h). To further quantify infection progression, we measured changes in host cell area over time, attributed to accumulation of auxosomes on the host surface, given cell size limitations imposed by the frustule (Fig. 1b,f,i). Indeed, diatom death at 48 h coincided with a peak in cell area (Fig. 1i). This phenotyping assay enabled precise inference of infection establishment timing (Fig. 1j), calculated as the point at which cell size increased by 10–15% of the initial value. *Pirsonia* infections occurred rapidly and synchronously, with all diatoms infected between 24 and 48 h after infection. By 96 h, all diatoms died, whereas zoospore numbers increased exponentially following differentiation from released auxosomes (Fig. 1k). Overall, live-cell time-lapse imaging enabled quantitative, single-cell tracking of *Pirsonia* infections, revealing a life cycle defined by parasite establishment and development, host consumption and mortality and zoospore proliferation, within 2 days. These infection dynamics highlight the capacity of *Pirsonia* to rapidly impact diatom population structure, a feature probably important in diatom bloom dynamics.

### The *P. diadema* genome reveals distinct lineage-specific innovations

**Fig. 2:**
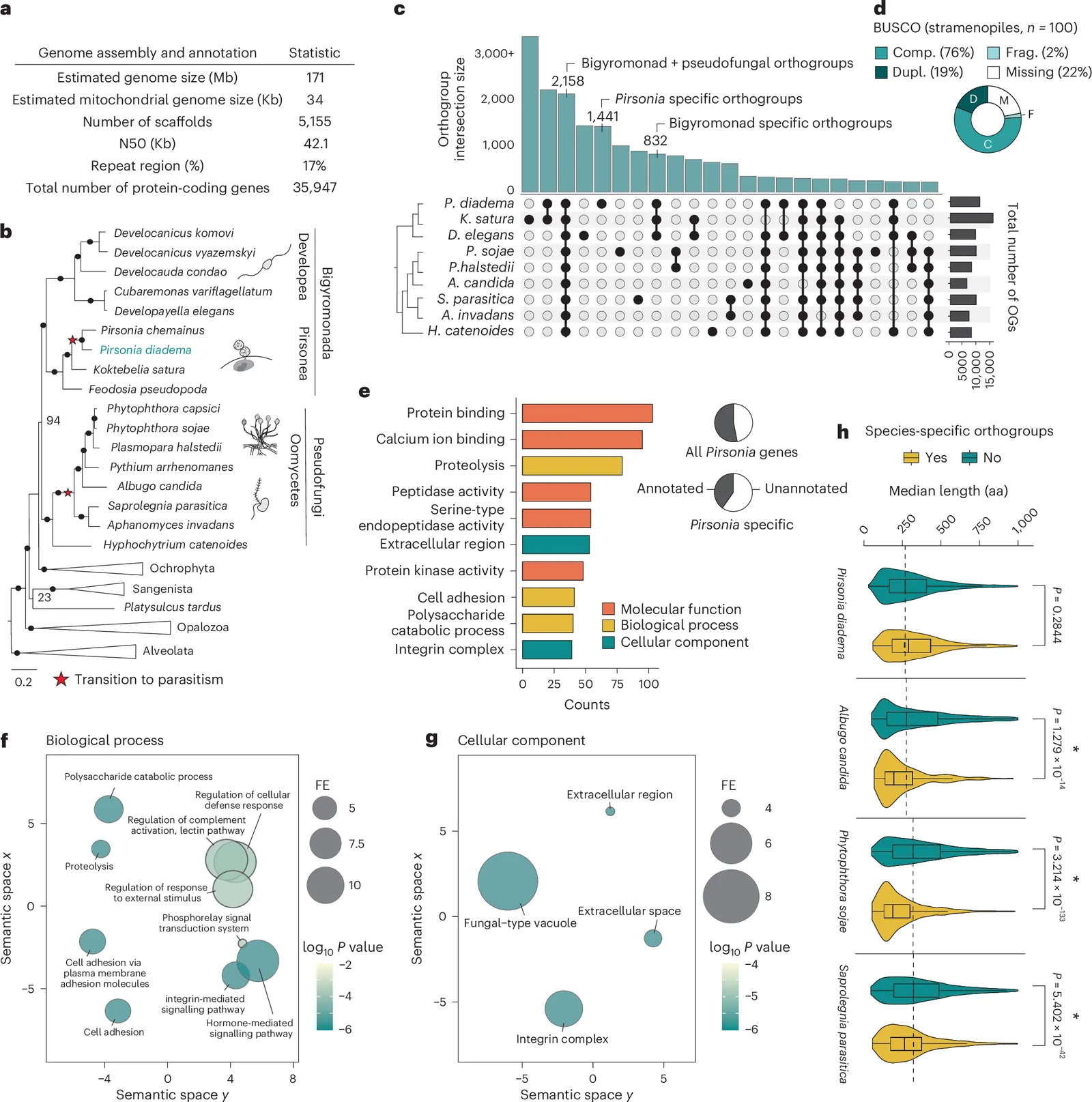
Comparative genomics of *P. diadema*. **a**, A summary of the *P. diadema* genome assembly and annotation statistics. **b**, The maximum likelihood tree of stramenopiles (with alveolate outgroup) generated from 242 genes using LG + C40 + F + G4 substitution model implemented in IQ-Tree. Support was obtained from 100 standard NPB and 1,000 UFB. Black circles represent NPB/UFB values greater than 95%, and transitions to parasitism are indicated. **c**, An upset plot displaying the shared and unique orthogroups across the Pseudofungi. Each row represents a pseudofungal species. Black circles and vertical lines between the rows represent the intersection of orthogroups. The cladogram (left) illustrates the phylogenetic relationships between species, and the bar plot (right) indicates the total number of orthogroups in each species. OGs, orthogroups. **d**, A doughnut chart displaying a summary of complete (Comp.), fragmented (Frag.), duplicated (Dupl.) and missing BUSCO genes in *P. diadema* genome on the basis of the stramenopile_odb10 lineage dataset. **e**, A bar plot displaying the most frequent GO terms found in the *P. diadema* genome annotations on the basis of counts. The colours indicate whether the GO term is a molecular function (red), biological process (yellow) or cellular component (green). The pie charts (top) display the proportion of *P. diadema* orthogroups that have GO annotation and (bottom) the proportion of *P. diadema*-specific orthogroups that have GO annotations. **f**,**g**, The scatter plots displaying enriched GO biological process (**f**) and cellular component (**g**) terms in *P. diadema* orthogroups annotations. Semantic similarity was determined using REVIGO and statistical significance was assessed using two-sided permutation tests (*P* < 0.01, *n* = 10^7^). FE, fold enrichment. **h**, Violin plots comparing protein length distributions in species-specific (yellow) and non-species-specific (teal) orthogroups in *P. diadema* and in three oomycete species. The horizontal dashed line marks the median length of non-species-specific orthogroups. The box plots show the median, interquartile range and whiskers extending to 1.5× the interquartile range. *P* values were calculated using two-sided Welch two-sample *t*-tests. Asterisks denote statistical significance. Source data

To understand the genetic basis of *Pirsonia*’s rapid and highly virulent life cycle, we characterized its genomic repertoire by generating a de novo draft genome using PacBio HiFi long-read sequencing. After stringent contamination removal (Methods), we assembled a 171-Mb genome of 5,155 scaffolds, with a scaffold N50 of 42.1 kb using Hifiasm^28^ (Fig. 2a). Gene annotation identified 35,947 protein-coding genes with 78% Benchmarking Universal Single Copy Orthologues (BUSCO) completeness (Fig. 2d). Together, these statistics confirm the quality of the *P. diadema* genome, which is, to our knowledge, the only genome sequenced so far from Bigyromonada.

To confirm the phylogenetic placement of *P. diadema*, we generated a multi-gene phylogeny of Stramenopiles (Fig. 2b and Supplementary Table 2). As expected, our analyses placed *P. diadema* within Bigyromonada, the sister group to the Pseudofungi, which contains the oomycetes and hyphochytriomycetes^20^, consistent with 18S ribosomal RNA gene phylogenies^23^. Oomycetes include major terrestrial and aquatic parasites that are predominantly filamentous osmotrophs with a brief flagellate zoospore stage^29^. By contrast, bigyromonads are mainly free-living heterotrophic flagellates^30^. Interestingly, *Koktebelia*, a free-living eukaryovorous predator, is the closest sister genus to *Pirsonia*, which is nested among other free-living bigyromonads^30^. Overall, this phylogeny supports *Pirsonia* as an independent transition to parasitism, alongside at least three distinct transitions within oomycetes^31,32^. These findings underscore the multiple, convergent origins of parasitism across Pseudofungi and Bigyromonada.

Given their parallel parasitic origins, we compared the gene repertoires of *Pirsonia* with other parasitic and free-living Pseudofungi. We clustered representative pseudofungal proteomes into 98,614 orthologous groups using OrthoFinder^33^ (Fig. 2c and Supplementary Table 3). Overall, bigyromonads exhibit more group-specific and species-specific orthogroups than oomycetes, suggesting greater gene-content divergence and highlighting limited sampling of bigyromonad diversity. *Pirsonia* shares most orthogroups with *K. satura* and other bigyromonads. Moreover, *Pirsonia* possesses many species-specific orthogroups (1,441). To explore their functions, we examined their annotations and found that only 40% had functional assignments, indicating many predicted proteins with unknown functions (Fig. 2e). Nonetheless, annotated *Pirsonia*-specific orthogroups were enriched in cell adhesion, cellular breakdown (proteolysis and polysaccharide catabolysis), signalling and regulation of cellular responses (Fig. 2e,f), processes potentially related to infectivity. In agreement, cellular components such as extracellular space, including proteins involved in integrin complexes, were strongly enriched. This raised the hypothesis that these proteins may represent effectors secreted by *Pirsonia* or cell surface proteins involved in host interactions (Fig. 2g). In oomycetes, effectors, such as proteins carrying an RxLR N-terminal motif^34^, are typically short and rapidly evolving. However, *Pirsonia*-specific proteins were not significantly shorter than the rest of the proteome (Fig. 2h). These differences may reflect different evolutionary pressures imposed by host immune systems, specifically between single-celled diatom hosts and multicellular hosts. These results demonstrate that beyond their morphological differences, *Pirsonia* and oomycetes genomes evolved under divergent evolutionary trajectories.

### Time series transcriptomics identify infection-associated genes in *P. diadema*

**Fig. 3:**
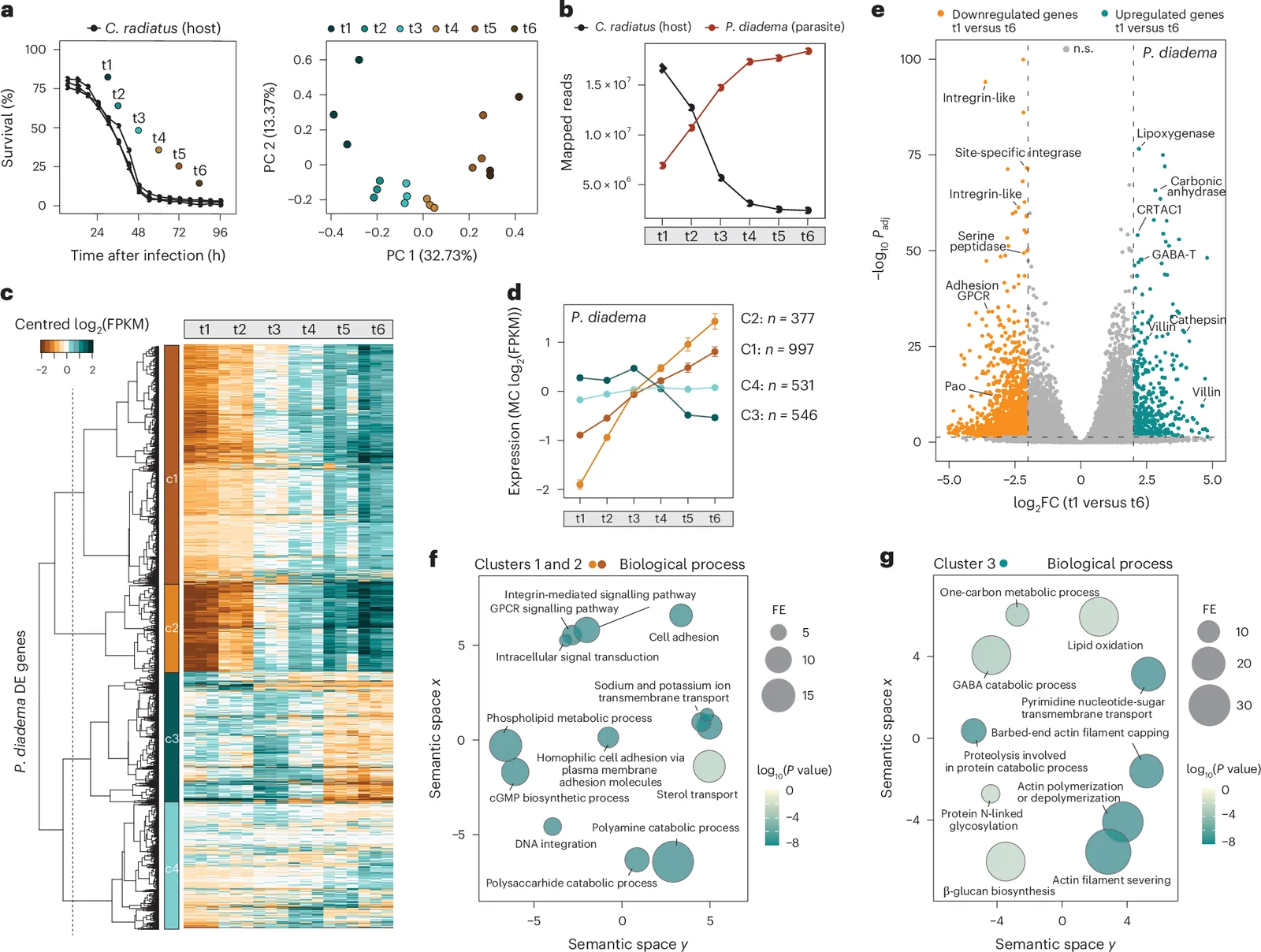
Time-resolved dual transcriptomics of *P. diadema* infection. **a**, The survival curve and PCA of samples. Left: a line plot displaying percent host survival over time since infection (in h), with sampled timepoints labelled as t1 through t6. Right: the PCA of RNA-seq reads, with each point representing a sample (in triplicate) and coloured according to its respective timepoint. **b**, A line plot of mapped RNA-seq reads for *C. radiatus* (host) and *P. diadema* (parasite) across sampled timepoints (t1–t6) after infection. **c**, A clustered heat map of 2,452 DE genes in *P. diadema* with a >4-fold change at *P* < 0.001 across sampled timepoints (t1–t6). Rows represent DE genes, and columns represent samples (and replicates). Expression values are shown as centred log_2_(fragments per kilobase of transcript per million (FPKM)), where FPKM-mapped reads values were log_2_-transformed and mean-centred (MC) for each gene across samples. The colour gradient shows relative expression changes: blues indicate upregulated genes, and browns indicate downregulated genes. Hierarchical clustering (cladogram) highlights the four main expression profiles labelled as c1–c4. **d**, A line plot illustrating the average expression patterns of the four main DE gene clusters in *P. diadema*. The number of genes in each cluster is displayed on the right. Expression is represented as mean-centred log_2_-transformed FPKM values. Error bars indicate standard deviation. **e**, The volcano plot of significantly differentially expressed genes in t1 compared with t6. The *y* axis corresponds to adjusted −log_10_ *P* values (*P*_adj_) and the *x* axis the log_2_ fold change in t1 compared with t6. Each point represents a gene, with orange points indicating genes that are downregulated in t1 relative to t6, and blue indicates genes that are upregulated in t1 relative to t6. Grey represents genes with no significant difference. **f**, A scatter plot displaying enriched GO biological process terms in *P. diadema* DE clusters 1 and 2. **g**, A scatter plot displaying enriched GO biological process terms in *P. diadema* DE clusters 3. For both plots, semantic similarity was determined using REVIGO, and statistical significance was assessed using two-sided permutation tests (*P* < 0.01, *n* = 10^7^).

To explore genetic contributions to the *Pirsonia* parasitoid lifestyle, we investigated gene expression changes during infection progression and life cycle transitions. We sequenced bulk *Coscinodiscus radiatus* cultures at six timepoints (t1–t6) following infection, encompassing key stages along the diatom survival curve (Fig. 3a). Principal component analysis (PCA) revealed tight clustering of biological replicates, with PC1 accounting for 32.7% of total variance, probably reflecting temporal separation of samples. To confirm that RNA sequencing (RNA-seq) recapitulated infection progression, we mapped each timepoint to a combined host–parasite reference transcriptome, revealing an inversion in host- and parasite-derived reads over time (Fig. 3b). This pattern is consistent with the *Pirsonia* life cycle, where early timepoints are dominated by active infections (t1–t3), whereas later timepoints are dominated by proliferating *Pirsonia* zoospores (t4–t6).

To identify differentially expressed (DE) genes across infection stages, we performed differential expression analysis using DESeq2^35^. We identified 2,452 genes in *Pirsonia* with a >4-fold change (*P* < 0.001) (Fig. 3c and Supplementary Table 4). Hierarchical clustering grouped these genes into four co-expression clusters (Fig. 3c and Supplementary Table 4), further categorized into three major expression patterns (Fig. 3d). Clusters 1 and 2 (1,334 genes) were highly upregulated in later timepoints (t4–t6), representing FMCs and zoospore-associated genes. By contrast, Cluster 3 (546 genes) was upregulated in early timepoints (t1–t3), suggesting association with active infections, including auxosome and intracellular trophosome formation. Cluster 4, which contained genes with mixed expression patterns across timepoints, was excluded from subsequent analyses.

To identify key genes differentiating t1 (trophosomes and auxosomes) and t6 (FMCs and zoospores), we examined the most significantly DE genes and found that most were unannotated predicted proteins, consistent with the high proportion of unknown genes in the genome (Fig. 2e). Among annotated genes, those upregulated in t1 and downregulated in t6 included cytoskeletal modifiers (for example, villin) and protein degraders (for example, cathepsin), whereas cell adhesion-related genes (for example, integrin alpha-like genes and adhesion G-protein-coupled receptors (GPCRs)) were upregulated in t6 and downregulated in t1. Overall, these findings indicate that early and late infection stages of *Pirsonia* exhibit distinct transcriptional profiles linked to the two main parasitoid cell types, auxosomes–trophosomes and infective flagellates.

To explore functions of DE genes across parasitic stages, we performed a Gene Ontology (GO) analysis (Fig. 3f,g). Enriched biological processes in the zoospore-associated clusters (clusters 1 and 2) included adhesion proteins and GPCR- and integrin-mediated signalling components, suggesting that *Pirsonia* flagellates may facilitate host recognition and attachment through membrane protein interactions or frustule binding. Moreover, processes related to ion homeostasis, such as sodium–potassium ion transport, might contribute to host membrane remodelling, alongside host membrane macromolecule degradation via polysaccharide and polyamine catabolism (Fig. 3f). By contrast, genes upregulated during active infection stages (cluster 3) were enriched in biological processes such as cytoskeletal genes functioning in actin polymerization, depolymerization, severing and capping (Fig. 3g). Other enriched functions in cluster 3 were associated with protein degradation (for example, proteolysis) and metabolite utilization (for example, one-carbon metabolism, lipid oxidation and pyrimidine nucleotide-sugar transport). This indicates that trophosomes may use these genes to facilitate nutrient uptake from hosts. In addition, GABA (γ-aminobutyric acid) catabolism enrichment suggests that *Pirsonia* may influence host cellular responses via interference with GABA receptor-mediated calcium signalling, a pathway implicated in diatom stress responses^36^. Together, these data provide insights into the molecular mechanisms by which *Pirsonia* infects its host, revealing distinct strategies involving attachment and host exploitation.

### A highly expanded integrin-like gene family in *Pirsonia*

**Fig. 4:**
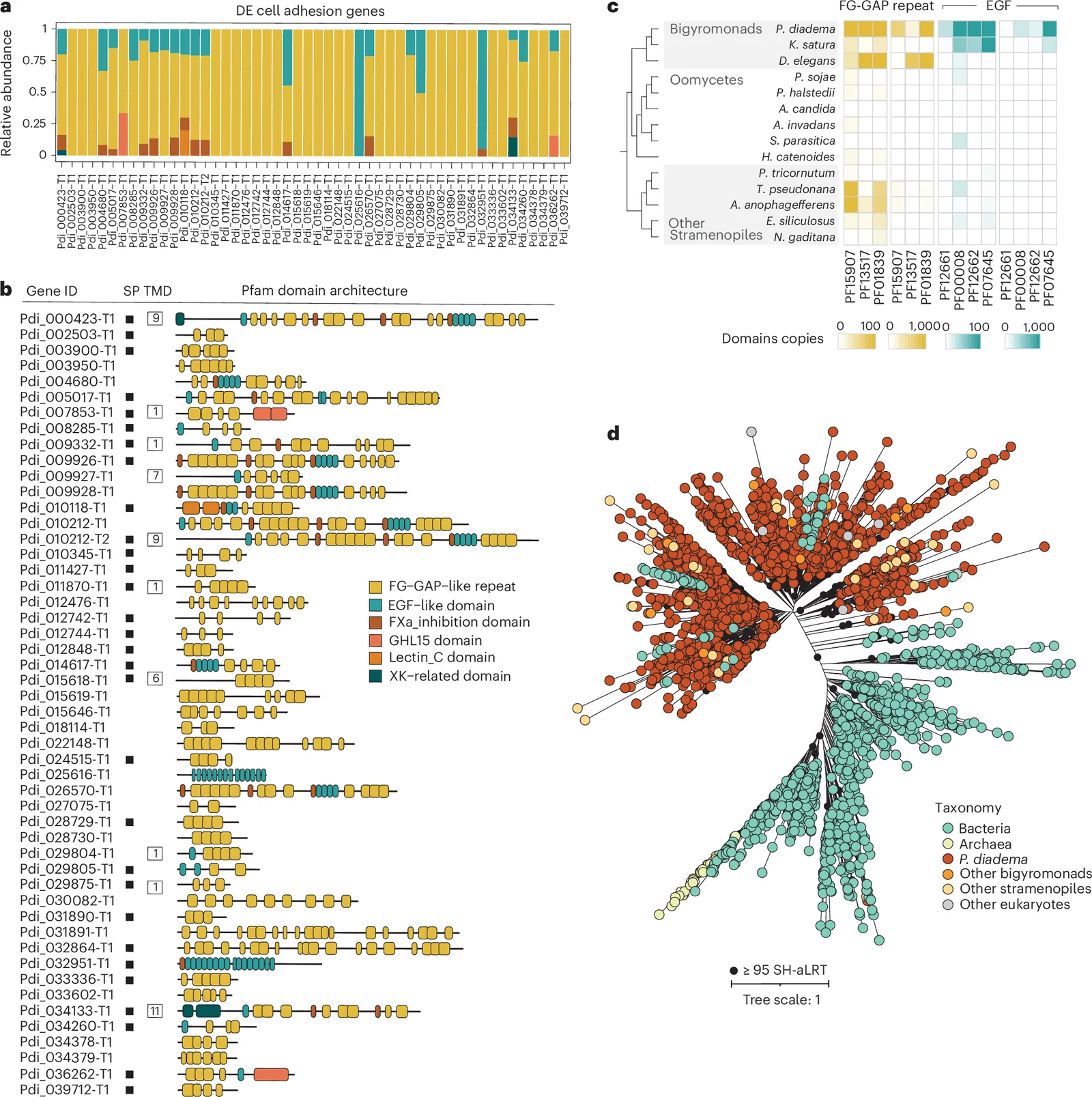
Analysis of integrin-like gene family in *P. diadema*. **a**, The relative abundance of Pfam protein domains found on DE cell adhesion genes. **b**, The protein domain architecture of DE cell adhesion genes. The presence of a signal peptide (SP) is denoted by a black square. TMDs are shown in white squares with the number of TMDs written inside. In **a** and **b**, domains are coloured by Pfam domain (with FG-GAP and EGF terms collapsed). The colour key is shown in **b** with Pfam domain labels. **c**, A heat map showing the number of FG-GAP (yellow) and EGF (blue) domains found in a reference set of stramenopile proteomes. The number of domains per proteome is shown in two scales: up to 100 and 1,000. The Pfam domain IDs are labelled below. **d**, The maximum likelihood phylogeny of FG_GAP_3 domain constructed in IQ-Tree. Support was obtained using 1,000 SH-aLRT. Bootstraps greater than 95% are shown as black circles. Tips are collapsed and coloured by species. Full tree can be viewed via iTOL^96^.

Given the enrichment of cell adhesion and integrin GO terms in both *Pirsonia* genome annotations and upregulated genes in zoospores, we examined their diversity and evolutionary history. Although integrins have fundamental roles in many metazoan physiological processes, they are not broadly found across eukaryotes^37^. All 51 DE *Pirsonia* cell adhesion genes contained FG-GAP repeats and EGF domains, similar to domains found in metazoan integrin-α and integrin-β (Fig. 4a). These proteins vary widely in length (~360–2,680 amino acids) and in FG-GAP and EGF domain numbers (Fig. 4b and Supplementary Table 5), in contrast to canonical metazoan integrins, which are generally 700–800 amino acids and contain either seven FG-GAP repeats (integrin-α) or four EGF repeats (integrin-β). Moreover, *Pirsonia* integrin-like proteins contain domains absent from metazoan integrins, such as FXa inhibition, glycoside hydrolase family 15, carbohydrate-binding C-type lectin and sodium transport XK-related domains (Fig. 4a). Furthermore, 60% of *Pirsonia* integrins were predicted to have signal peptides using DeepTMHMM v1^38^ and DeepLoc v2.1^39^, with 20% also containing transmembrane domains (TMDs) (Fig. 4b and Supplementary Table 5). Notably, several *Pirsonia* integrins possess multiple TMDs, unlike metazoan integrins, which function as single-pass transmembrane proteins. Collectively, these findings indicate that *Pirsonia* integrin-like genes are structurally and functionally distinct from canonical metazoan integrins.

To investigate their evolutionary history, we performed hidden Markov model (HMM) searches for FG-GAP repeat- and EGF domain-containing proteins across diverse stramenopiles (Fig. 4c). Although we identified proteins containing these domains in oomycetes and other stramenopiles, they are significantly more abundant in *Pirsonia*, which has 445 annotated integrin-like genes containing thousands of FG-GAP and EGF domains (Fig. 4c). Introns within these genes indicate they are unlikely to originate from bacterial contamination (Supplementary Table 5). Notably, bigyromonads such as *K. satura*, the sister group to *Pirsonia*, also contain many EGF domain-containing proteins but fewer FG-GAP domain proteins, whereas *D. elegans*, another related bigyromonad species, shows the opposite pattern. This suggests that genes encoding these domains were present in the last common stramenopile ancestor and expanded significantly in bigyromonads, particularly *Pirsonia*, potentially as an adaptation to diatom parasitism.

To investigate the evolutionary history of FG-GAP-like repeat-containing proteins, we expanded our search to a balanced set of taxonomically diverse reference proteomes across the tree of life (Methods) and constructed a maximum likelihood phylogeny (Fig. 4d). We identified orthologues primarily from diverse bacterial lineages, photosynthetic ochrophytes (stramenopiles) and other eukaryotic groups, including cryptomonads (*Guillardia*), haptophytes (*Diacronema*) and green algae (*Ostreobium*). Eukaryotic sequences formed a distinct clade, separate but related to a large assemblage of bacterial sequences. Given current genomic datasets, this topology may reflect either an ancient eukaryotic origin followed by extensive loss across most eukaryotic lineages or horizontal gene transfer from bacteria followed by a lineage-specific expansion in *Pirsonia*. Resolving these scenarios will require deeper genomic sampling of undersampled eukaryotic lineages and functional characterization of these FG-GAP-like proteins.

### Cytoskeletal dynamics are required during *Pirsonia* infection

**Fig. 5:**
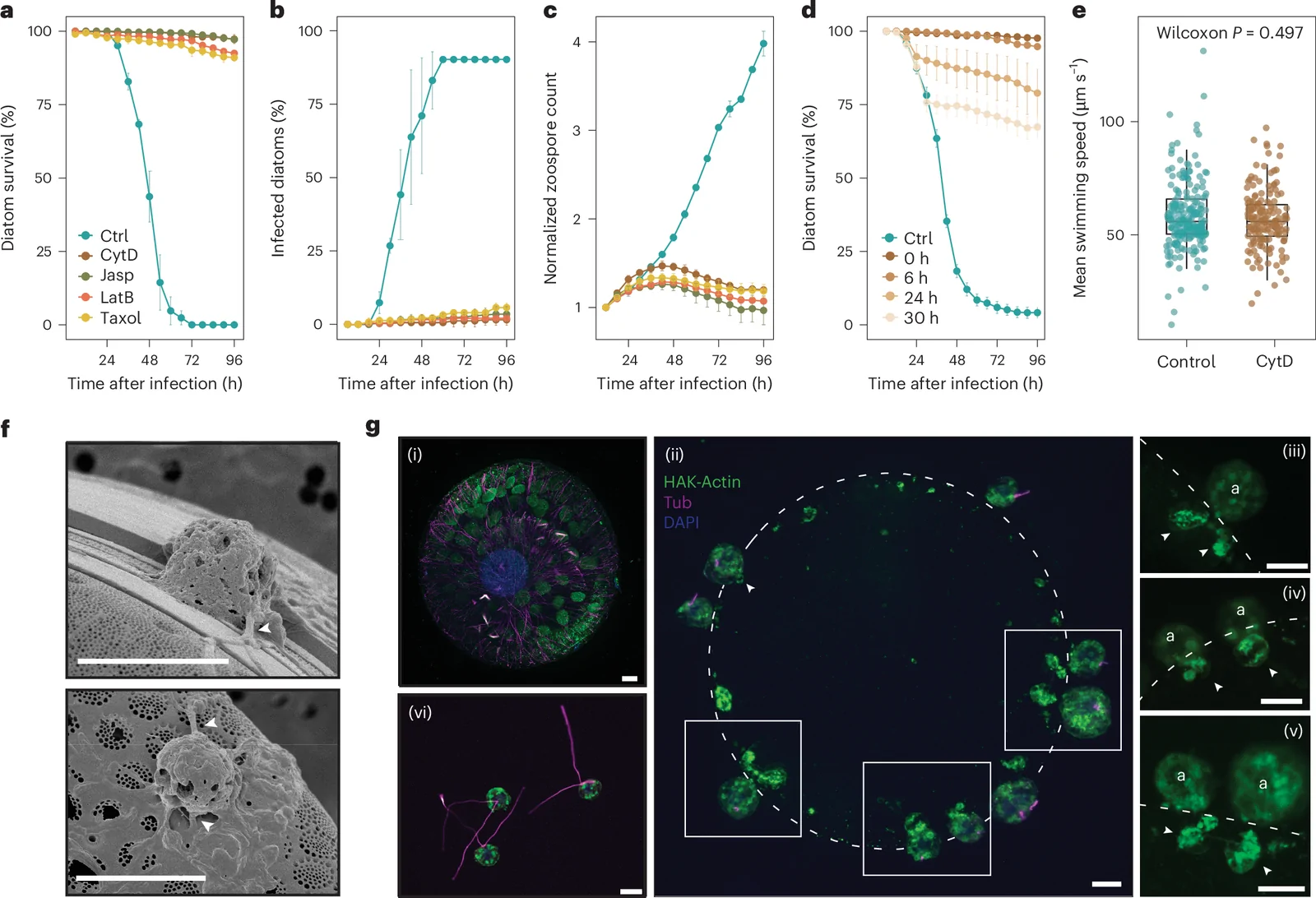
Cytoskeletal dynamics underpin infection processes in *Pirsonia*. **a**, The percentage of diatom survival over time after infection, with survival defined as CTCF remaining above 50% of the well-averaged baseline. Ctrl, control; CytD, cytochalasin. **b**, The percentage of diatoms infected over time, calculated by a 15% increase in cell area. **c**, The zoospore counts normalized to initial values for each well, measured throughout the infection. **d**, The percentage of diatom survival following the time-dependent addition of cytochalasin D. Top: the automated live-cell HCS of *P. diadema* infecting *C. granii* under cytoskeletal perturbations. Data in **a**–**d** are presented as mean ± s.d. from independent biological replicate wells per condition. **e**, The mean swimming speed of *P. diadema* zoospores in dimethylsulfoxide (DMSO) control and cytochalasin D-treated conditions over 18 h. Points represent individual zoospore tracks pooled from nine biological replicate wells per condition (DMSO control, *n* = 198 tracks; cytochalasin D, *n* = 163 tracks). The box plots show the median, interquartile range and whiskers extending to 1.5× the interquartile range. Statistical significance was assessed using a two-sided Wilcoxon rank-sum test on track measurements (*P* = 0.497). Bottom: the visualization of parasite-associated cytoskeletal structures. Images in **f** and **g** are representative of observations from at least three independent infections with similar results. **f**, The SEM of *P. diadema* auxosome attached to the diatom frustule. White arrows indicate thin, pseudopodia-like projections at the host–parasite interface. Scale bars, 5 µm. **g**, The U-ExM (expansion factor 4.1×) with immunostaining for F-actin (HAK-actin, green), microtubules (12G10 anti-α-tubulin, magenta) and DNA (DAPI, blue). Maximum intensity projections of (i) uninfected *C. granii*, (ii) late-stage *P. diadema* infection (dotted line outlines emptied frustule), (iii–v) magnified views of auxosomes with HAK-actin only, **a**, connected to intracellular trophosomes and (vi) *P. diadema* zoospores. White arrows highlight actin in trophosome compartments beneath auxosome attachment sites. Expanded scale bars, 20 µm, biological scale bars, 5 µm. Source data

In metazoans, integrins link the extracellular matrix and intracellular actin cytoskeleton^37^. Given the convergence in protein domains and transmembrane localization of *Pirsonia* FG-GAP- and EGF domain-containing genes with canonical integrins, together with the enrichment of actin-related functions in auxosomes–trophosomes (Fig. 3g), we hypothesized that *Pirsonia* infection depends on cytoskeletal dynamics. To test this, we treated infected diatoms with three actin-disrupting agents and monitored infection progression using live-cell time series high-content imaging (Fig. 5a–c and Extended Data Fig. 2). Cytochalasin D, which caps actin filaments and blocks polymerization, latrunculin B, which sequesters actin monomers, and jasplakinolide, which stabilizes filaments, all impaired infection under standard working concentrations. Treated cells showed complete diatom survival (Fig. 5a), no successful infections (Fig. 5b) and stable zoospore numbers (Fig. 5c), indicating that dynamic actin remodelling is essential for successful *Pirsonia* infections. Furthermore, time-dependent treatment with cytochalasin D at 6, 24 and 30 h after infection rescued diatoms and inhibited the infection cycle (Fig. 5d), demonstrating that actin is required for both infection initiation and progression. Next, to assess whether actin disruption impairs infection by affecting zoospore motility, we treated *Pirsonia* zoospores with cytochalasin D and observed no significant change in zoospore motility relative to controls, even after 18 h (Wilcoxon rank test, *P* = 0.497; Fig. 5e). These results indicate that actin disruption is unlikely to inhibit infection by impairing zoospore motility and instead support a specific requirement for actin dynamics during auxosome attachment and trophosome function, consistent with its continued role throughout the infection.

Finally, we evaluated whether infection inhibition reflected indirect effects on host physiology by assessing host health in the presence of cytochalasin D at infection-inhibiting concentrations. We detected no differences in chlorophyll autofluorescence or survival, indicating that host viability and cellular function were not compromised (Extended Data Fig. 3). Consistently, diatoms pretreated with cytochalasin D for 12 h, followed by drug washout before infection, were infected normally by *Pirsonia* (Extended Data Fig. 3), further supporting parasite-specific reliance on actin dynamics rather than an indirect host effect. For comparison, we treated infected cultures with taxol (paclitaxel), a microtubule-stabilizing agent expected to interfere with zoospore function owing to the well-established role of microtubules in flagellar motility. As anticipated, taxol impaired infection (Fig. 5a–c and Extended Data Fig. 2), suggesting that distinct cytoskeletal systems, including microtubule-based processes in zoospores and actin-based dynamics in auxosomes–trophosomes, may act at different stages to complete the *Pirsonia* infection cycle.

To understand cytoskeletal roles during infection, we visualized host and parasite cytoskeletal organization using high-resolution imaging. We first examined parasite attachment sites by SEM and observed pseudopod-like extensions at the interface between *Pirsonia* auxosomes and the diatom frustule, suggestive of actin-based attachment (Fig. 5f). Next, to resolve cytoskeletal architecture within trophosomes, we performed ultrastructure expansion microscopy (U-ExM) combined with immunolabelling^40,41^. U-ExM was performed on uninfected diatoms, infected diatoms and *Pirsonia* zoospores to resolve cytoskeletal organization across infection stages, with roughly fourfold expansion enabling detailed visualization of proteinaceous structures, F-actin and microtubules (Fig. 5g and Extended Data Fig. 4). In uninfected *C. granii* cells (top left), actin signal was predominantly associated with chloroplast regions, whereas microtubules formed a network reflecting the cell’s overall symmetry (Extended Data Fig. 4). To visualize parasite-associated structures, we focused on late-stage infections in which host-derived actin signal was minimized owing to the complete degradation of host cellular components. Under these conditions, clear actin signal was detected in auxosomes and intracellular trophosomes located below auxosome attachment sites (Fig. 5g, centre and left), highlighting actin as a prominent structural component of the intracellular stage. Discrete tubulin puncta were also observed on late-stage auxosomes, consistent with initiation of flagellar assembly before detachment and differentiation into FMCs (Fig. 5g, centre). In zoospores, actin was detected within the cell body, whereas microtubules localized prominently to the flagella. Together, these observations provide independent, stage-resolved evidence that *Pirsonia* deploys cytoskeletal systems in a context-dependent manner, with actin enriched at host attachment sites and within intracellular trophosomes and microtubules largely restricted to flagella.

## Discussion

Here, we establish a model pathosystem for investigating phytoplankton–parasite interactions, comprising the globally distributed, bloom-forming, environmentally important diatom *Coscinodiscus* and its parasitoid *P. diadema*. Together with phenotyping assays based on high-throughput live-cell imaging, this system offers a unique platform to dissect the molecular and cellular mechanisms underlying a marine host–parasite interaction. The rapidity of the *Pirsonia* life cycle and its capacity to cause total diatom population collapse in culture highlight how microbial parasites shape biogeochemical nutrient cycling similarly to marine viruses^42^.

The genome assembly of *P. diadema* (Fig. 2), a representative of the largely free-living bigyromonads, and transcriptomic identification of infection-associated genes provide a comparative system for examining parasitic evolution. Integrin-like proteins, identified as candidate variable surface proteins in *Pirsonia*, reveal a unique yet convergent hallmark of parasite genomes^43^. These uncharacterized proteins may function in surface adhesion or recognition of host-derived ligands, reminiscent of strategies used by other eukaryotic parasites such as *Plasmodium*^44^, trypanosomes^45^ and oomycetes^46^, although other parasitic species have co-opted alternative gene families for this function. Integrin expansion in *Pirsonia* suggests a lineage-specific adaptation to diatom parasitism, possibly mediating host recognition on the diatom frustule or facilitating entry into the host cytoplasm. Together, these findings emphasize surface protein diversification in parasite evolution across eukaryotes.

The susceptibility of *Pirsonia* to actin inhibitors, surface pseudopodia visualization (Fig. 5f) and detection of actin within trophosomes (Fig. 5g) suggest that auxosome attachment and trophosome formation may be actin-dependent, as hypothesized in early studies^47^. Although actin involvement is unsurprising given its central role in eukaryotic cell biology, several alternative infection strategies were plausible, including actin-independent trophosome formation, hyphal growth rather than pseudopodial invasion or host digestion via secretion-based osmotrophy typical of fungal and oomycete parasites. Actin-dependent invasion strategies have evolved independently across diverse parasitic lineages; for example, apicomplexans use the actin-myosin-based glideosome for host invasion^48^, whereas the chytrid fungus *Batrachochytrium* dynamically remodels its actin cytoskeleton during infection^49^. Pseudopodia-based adhesion could also facilitate firm host attachment in the turbulent surface waters inhabited by *Pirsonia*. Notably, a recent taxonomic study showed that *Pirsonia* relatives include numerous predatory species that engulf and digest eukaryotic prey using pseudopodia^30^. However, auxosomes and trophosomes remain unique to *Pirsonia* parasitoids. Thus, although phagocytosis and pseudopodia are probably ancestral traits linked to predation, *Pirsonia* has co-opted and repurposed these mechanisms for parasitism. This trait blurs the distinction between parasitism and predation, positioning *Pirsonia* as a key group for studying evolutionary shifts in trophic strategies and the origins of parasitism. Expanded genomic sampling of *Pirsonia* species and their free-living relatives, alongside detailed cell biology, will be necessary to uncover the molecular innovations underpinning this transition.

**Extended Data Fig. 1:**
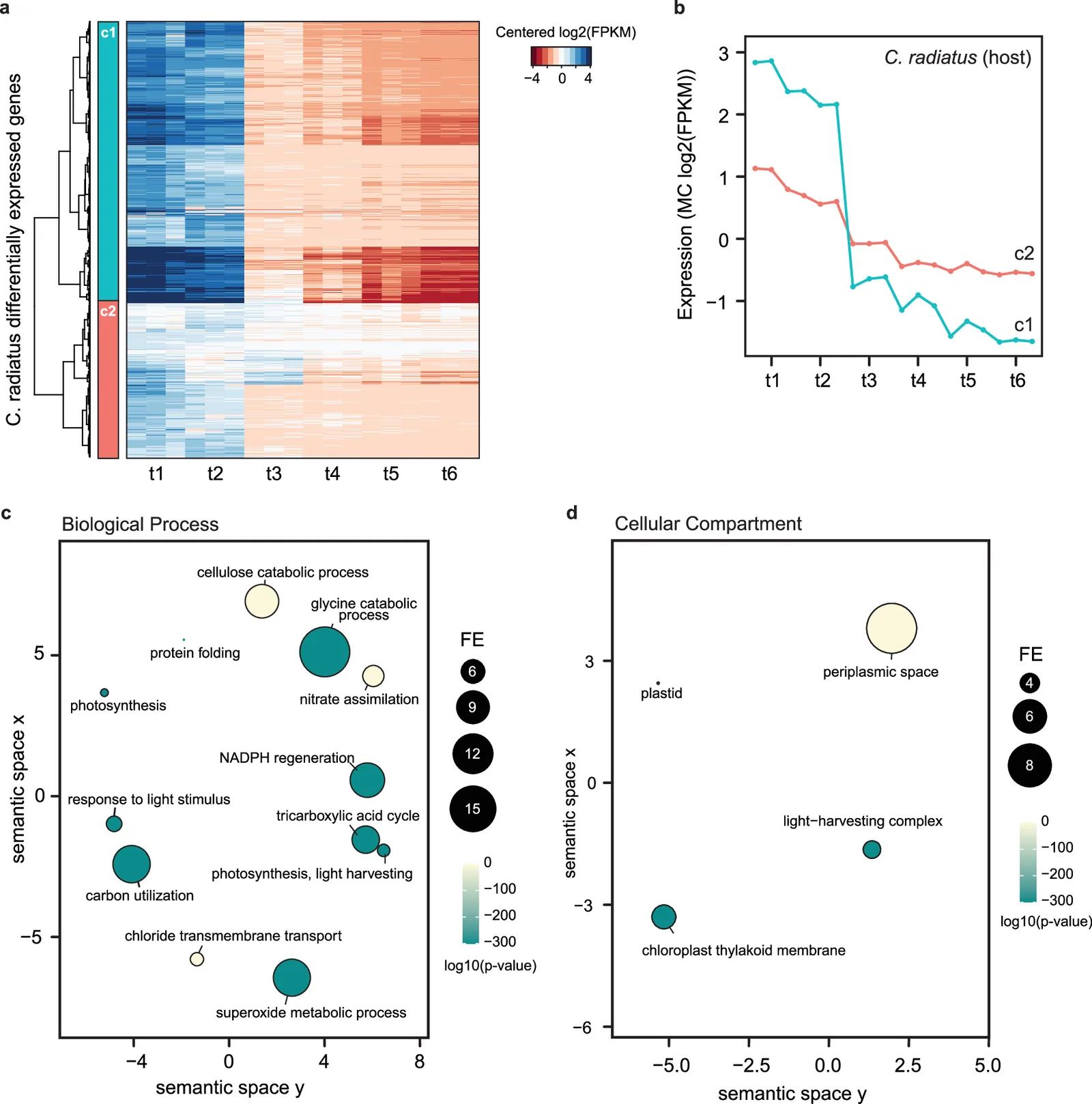
Transcriptomics of C. radiatus during Pirsonia infection. **a**, Clustered heatmap of 2,057 differentially expressed (DE) genes in C. radiatus with >4-fold change at p < 0.001 across the sampled timepoints post-infection (t1–t6). Rows represent DE genes, and columns represent samples (and replicates). Expression values are shown as centered log_2_(FPKM), (where Fragments Per Kilobase of transcript per Million mapped reads were log_2_-transformed and mean-centered for each gene across samples). Colour gradient shows relative expression changes: blues indicate upregulated genes and reds indicate downregulated genes. Hierarchical clustering (cladogram) highlights the two main expression profiles labelled as c1 and c2. **b**, Line plot illustrating the average expression patterns of DE gene clusters. Expression is represented as mean-centered log_2_-transformed FPKM values. **c**, **d**, Scatterplots displaying enriched GO biological process and cellular compartments terms in the DE genes, respectively. For both plots semantic similarity was determined using REVIGO and statistical significance was assessed using two-sided permutation tests p < 0.01, n = 107). Source data

**Extended Data Fig. 2:**
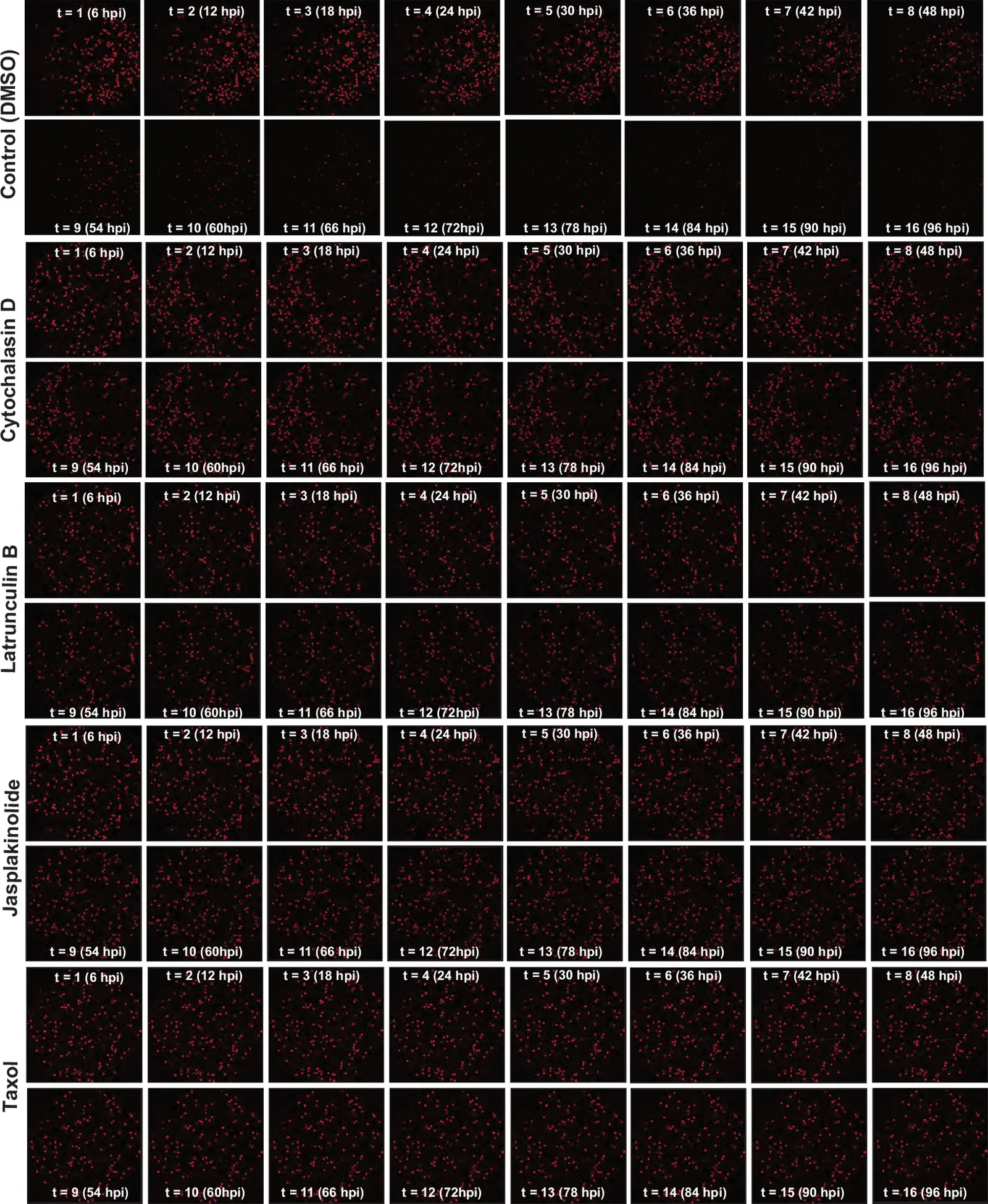
High-Content Screening (HCS) of Pirsonia infection with drug perturbations. Single well view of the Cy5 channel (chlorophyll autofluorescence). t = time point; hpi = hours post-infection. From top to bottom: control infection (DMSO), Cytochalasin D, Latrunculin B, Jasplakinolide, Taxol. Source data

**Extended Data Fig. 3:**
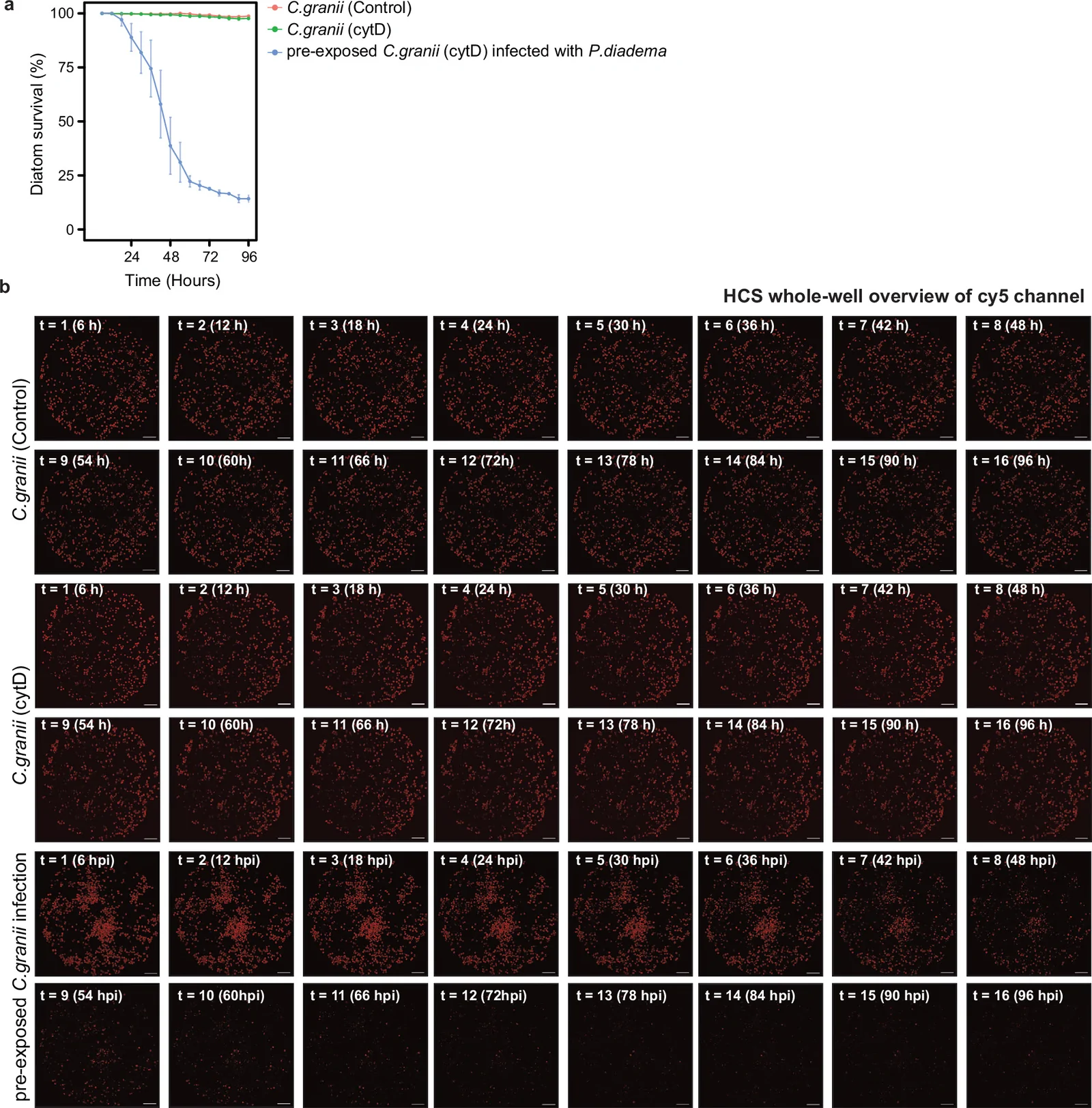
Host viability under drug conditions that impair parasite infection. **a**, Diatom survival curve calculated based on CTCF levels for the following conditions: pink, C. granii (control, in DMSO); green, C. granii treated with cytochalasin D (8 µM); blue, C. granii treated with cytochalasin D (8 µM) for 12 hours, washed, and then infected with P. diadema. All conditions were performed in three independent biological replicates; error bars represent standard deviation. **b**, High-Content Screening (HCS) of the above conditions. Images represent a single well view of the Cy5 channel (chlorophyll autofluorescence). t = time point, hpi = hours post-infection, h = hours. Scale bars = 500 µm.

**Extended Data Fig. 4:**
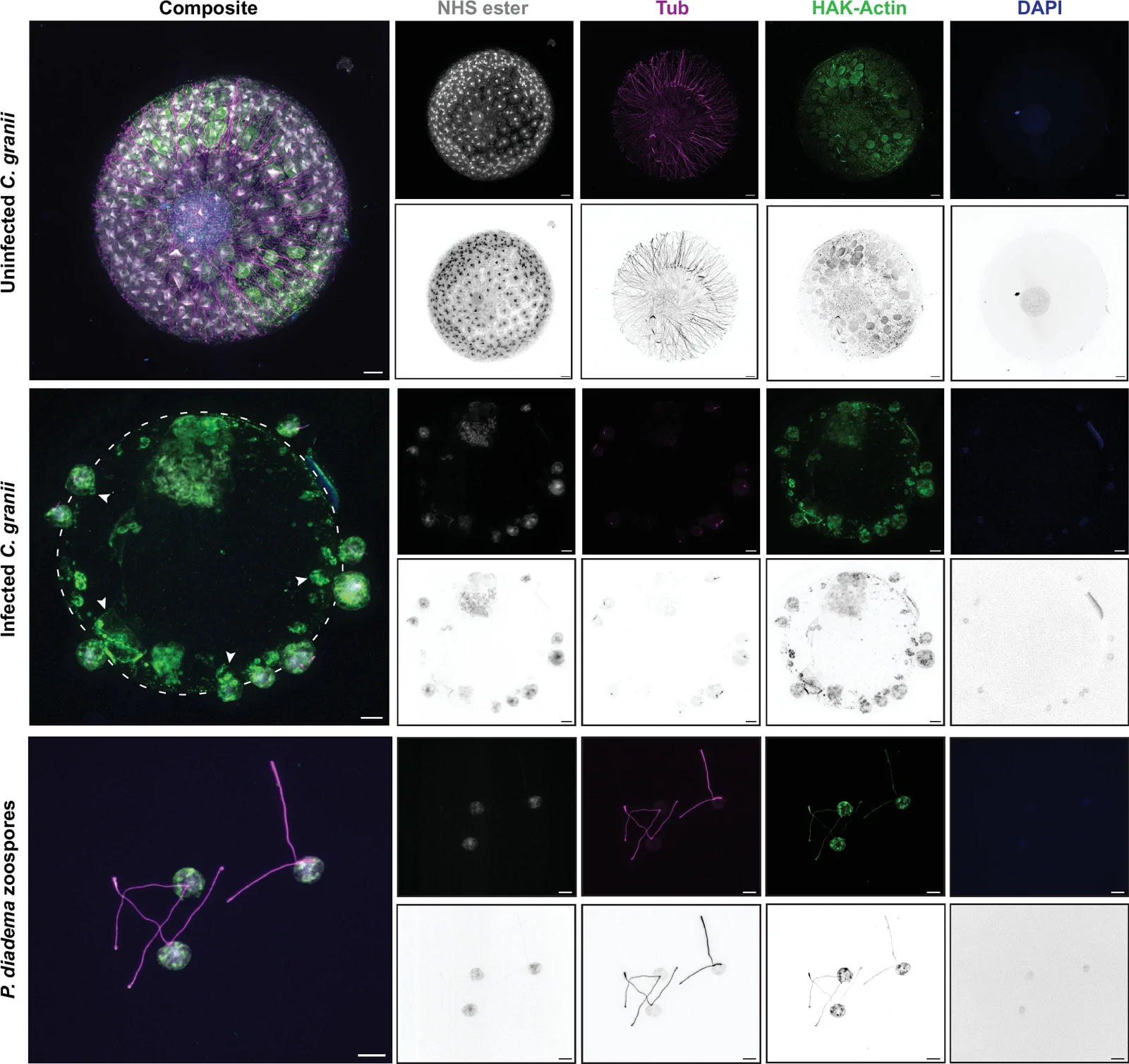
Ultrastructure Expansion Microscopy (U-ExM) inverted grayscale antibody staining. Maximum-intensity projection confocal fluorescence images were converted to grayscale and intensity-inverted, such that higher antibody signal appears darker. Top panel, Uninfected C. granii. Middle panel, P. diadema infecting C. granii at a very late stage of infection, where the host is non-viable. Bottom panel, P. diadema zoospores. NHS-ester (gray), F-actin (HAK-actin, green), microtubules (12G10 anti-α-tubulin, magenta), and DNA (DAPI, blue). Expanded scale bars = 20 µm; biological scale bars = 5 µm. Differential intensity in HAK-actin staining may result from asymmetric proximity to the coverslip, leading to increased signal due to light refraction with imaging depth.

Another key question about the *Pirsonia*–*Coscinodiscus* interaction is whether the diatom mounts an immune response and whether, in nature, parasitoids and hosts coexist, similar to the coevolutionary arms race between marine viruses and their hosts^42^. Our transcriptomic approach could not resolve the host transcriptional response to *Pirsonia* infection beyond signatures of cellular collapse and death (Extended Data Fig. 1 and Supplementary Table 6). Current evidence for pathogen-specific immune responses in diatoms remains limited. Diatoms appear to rely on stress-mediated defence pathways, including redox^50^ and nitric oxide signalling^36^ and programmed cell death^51^, which are well documented during biotic stress but are thought to act mainly at the population level, rather than through targeted pathogen recognition or effector-based defence mechanisms. In this context, the apparent lack of small effectors in *Pirsonia* (Fig. 2h) suggests that reduced immune pressure from its unicellular diatom host allows the retention of a broader, less specialized secretome consistent with its large, gene-rich genome. By contrast, parasites infecting multicellular hosts, such as the plant-infecting oomycete *Phytophthora sojae*, face intense immunological challenges and have evolved specialized effectors, including duplicated decoy effectors that neutralize host defences^52^. The absence of detectable host responses in our bulk RNA-seq may reflect methodological limitations, as subtle, transient or cell-specific responses are probably masked in population-level analyses. Single-cell transcriptomic and spatial proteomic approaches, increasingly applied across diverse host–parasite systems^53,54^, offer promising avenues to resolve fine-scale diatom responses and investigate Red Queen dynamics driving host–parasite coevolution.

By providing a detailed molecular and genomic investigation of a planktonic host–parasite interaction, this study highlights both current limitations and important avenues for future work. In particular, direct functional validation of the proposed roles of infection-associated genes such as integrins and actin will require genetic manipulation tools in *Pirsonia*, including targeted knockdowns or CRISPR-based approaches, as well as live-cell imaging of cytoskeletal regulators to test the hypothesized repurposing of ancestral predatory machinery. Moreover, these interactions occur within complex microbial communities, and growing evidence shows that diatoms engage in diverse biotic relationships with bacteria and other microorganisms^55,56^. Incorporating microbial players and environmental dynamics into this model will be essential to unravel host–parasite dynamics in marine ecosystems. Although the ecological importance of parasites in oceanic food webs is increasingly recognized^57^, key aspects of parasite-mediated phytoplankton mortality and its consequences remain largely unexplored. By establishing a model pathosystem, our study provides a critical foundation for elucidating the molecular and ecological mechanisms underlying marine parasitism.

## Methods

### Sampling and cultivation of host and parasite

*C. radiatus* and *C. granii* were isolated from marine plankton tows carried out at station L4 (50° 20′ 0.093″ N, 4° 08′ 0.915″ W) located southwest of Plymouth in the Western English Channel. To establish cultures, *Coscinodiscus* single cells were manually isolated using a microcapillary pipette, washed multiple times in autoclaved ASW (30 ppt) and propagated in F/2 + Si media (Guillard’s medium for diatoms). *P. diadema* was isolated from the net-tow sample at the Yongho Bay (35° 08′ 00″ N, 129° 06′ 55″ E) of Busan, Republic of Korea*. P. diadema* was cultivated by inoculating 20 ml of *C. radiatus* with 1 ml from a 4-day-old, infected culture. Both host and parasite cultures were maintained in a 12-h light–dark cycle at 18–21°C. As cryopreservation is not possible for these organisms, cultures must be maintained continuously and are therefore challenging to sustain long-term. The *C. radiatus* strain was subsequently lost; however, *C. granii* and *P. diadema* cultures are available from the authors upon request.

### SEM and confocal microscopy

For SEM, *C. radiatus* infected with *P. diadema* were fixed by adding 25% glutaraldehyde in artificial seawater (ASW) directly into a 3-day-old 20-ml culture followed by a 4-h incubation at 4 °C. Fixed cells were gently filtered onto a Whatman Nucleopore Track-Etched Membrane with a pore size of 0.8 µm. Cells on the filter were gently washed three times with Milli-Q water and dehydrated in a graded ethanol series (30%, 50%, 70%, 80%, 90% and 99%) with a 15-min incubation at each step. Cells were chemically dried with a 10-min incubation followed by an overnight incubation in 100% hexamethyldisilane. The filter was critical point-dried with CO_2_ and sputter coated before imaging.

Confocal microscopy was performed by concentrating 40 ml of infected diatoms via gravity filtration using a 40-μm pluriStrainer (pluriSelect). Cells were resuspended in 0.5 ml of ASW, and a 100-μl drop was placed on a poly-L-lysine-coated Poly-Prep glass slide (Sigma-Aldrich) for 1 h to allow sedimentation and attachment. Excess ASW was removed by gently tipping the slide and blotting the edges with filter paper. Cells were fixed in 250 μl of 4% (w/v) paraformaldehyde in ASW for 15 min at room temperature. The fixative was replaced with 250 μl ASW for a 1-min wash. Cells were then permeabilized in 250 μl PBS with 0.1% (v/v) Triton X-100 in ASW for 5 min at room temperature. After a second wash, Hoechst 33342 (Sigma-Aldrich, H3570) was added at a 1:500 (v/v) dilution for 60 min at room temperature. Following two additional washes, 7 μl of ASW was added to the slide, which was then covered with a coverslip and sealed with transparent nail polish to prevent evaporation. Imaging was performed on a Zeiss LSM-780 confocal microscope using 20× and Ph3 60× oil objectives. Exposure settings were kept constant, and images were acquired with ZEISS ZEN software and processed using FIJI (ImageJ v1.54)^59^.

### Environmental survey analysis

To investigate the presence and distribution of *Pirsonia* parasites, we used the publicly available Tara Oceans 18S V9 metabarcoding dataset, clustered into biologically meaningful OTUs using the Swarm algorithm with subsequent refinement via the MuMUs method^27^. 18S ribosomal RNA sequences of all known *Pirsonia* species were queried against the Tara Oceans V9 Swarm-MuMUs dataset using BLASTn^60^ (E-value ≤ 1 × 10⁻^15^). Hits with ≥90% sequence identity were retained and subjected to reciprocal BLASTn searches against the NCBI nt database^61^. For each OTU, the best hit was selected on the basis of the highest bitscore, with ties resolved by the lowest E-value. OTUs whose best hit was annotated as an ‘uncultured eukaryote’ were further evaluated by examining the taxonomy of their top ten hits. Final OTU assignments were restricted to hits with ≥97% sequence identity to a *Pirsonia* species and amplicon lengths ≥100 bp. Samples were considered positive for *Pirsonia* if they contained at least one *Pirsonia* OTU with a read abundance of ≥10.

### Time series HCS, drug assays and image analysis

*P. diadema* zoospores were harvested from infected *Coscinodiscus* cultures 4 days after infection. For time-resolved life cycle quantification and drug perturbation assays, *C. radiatus* or *C. granii* cells were put into 96-well plates and inoculated with *P. diadema* zoospores. Infections were imaged every 6 h for 96 h (16 timepoints) at 18 °C using automated high-content imaging platforms (either the ImageXpress Pico Automated Cell Imaging System (Molecular Devices), Zeiss Cell Discoverer 7 or Zeiss LSM800 inverted confocal microscope). Brightfield and Cy5 channels were acquired at 5× or 10× magnification. Whole-well tiled images were generated using CellReporterXpress (Molecular Devices) or Zeiss ZEN software. For cytoskeletal drug perturbation assays, infected *C. granii* cultures were treated with cytochalasin D (8 µM), latrunculin B (5 µM), jasplakinolide (3 µM) or taxol (5 µM). All drugs were prepared in DMSO, with a final DMSO concentration of 0.3% in all wells, including controls. For all drug treatments, dose–response assays were conducted and the lowest concentration that reproducibly impaired infection was used in subsequent experiments. For time-of-addition experiments, cytochalasin D (8 µM) was added to infected *C. granii* cultures at 0, 6, 24 or 30 h after infection. For washout experiments, *C. granii* cultures were pre-treated with cytochalasin D (8 µM) for 12 h at 18 °C, washed twice with F/2 media to remove the drug, inoculated with *P. diadema* zoospores and imaged as described above. Host-only control experiments were performed by treating *C. granii* cultures with cytochalasin D (8 µM) in the absence of *P. diadema* and imaged every 6 h for 96 h. All experiments were performed with three independent biological replicates (three wells per condition).

Image analysis was performed in FIJI (ImageJ v1.54)^59^ using the Labkit plugin for supervised segmentation. Measurements including integrated density (IntDen), mean fluorescence and cell area, were extracted per diatom cell across all timepoints. The same was done for zoospores. CTCF was calculated as: CTCF = IntDen − (area × background). Outliers in CTCF and area were removed using an interquartile range filter. Cells were tracked on the basis of their positional coordinates and only cells tracked across all timepoints were included in downstream analysis. Survival curves defined live cells as those maintaining CTCF above 50% of the well-averaged baseline. Infection dynamics were quantified by tracking individual cells and classifying them as infected if their area increased by ≥10–15% relative to baseline. To reduce false positives from transient shape changes, size trajectories were cross validated against the final size. Zoospore counts were filtered by area to exclude debris, averaged across replicates and normalized to the first timepoint. All analyses and visualizations were performed in R v2023.12.1 + 402 (ggplot2 and dplyr) and Python v3.13.3.

### Zoospore motility assays

*Pirsonia* zoospores were harvested on day 4 after infection by filtration through a 40-µm cell strainer. Zoospores (100 µl per well) were distributed into multi-well plates with cytochalasin D (8 µM final concentration; 0.4% DMSO control), with nine wells per condition. After 18 h incubation, time-lapse imaging was performed on a Zeiss Cell Discoverer microscope using a 5× objective (180 frames, 0.05-s interval). Image sequences were processed in FIJI (ImageJ v1.54)^59^ by median background subtraction and Gaussian blurring (*σ* = 1.5). Zoospore trajectories were extracted using TrackMate with a Laplacian-of-Gaussian detector (12-µm diameter, threshold 0.2), median filtering, subpixel localization and the Simple LAP tracker. Track data were analysed in R with only tracks ≥2.5 s in duration and ≥50 µm in displacement were included. To avoid pseudoreplication, swimming speed was summarized as the median per well, and statistical significance between conditions was assessed using a Wilcoxon rank-sum test.

### PacBio HiFi genome sequencing and assembly

A total of 1 l of dense *C. radiatus* cultures were infected with *P. diadema* zoospores. At 4 days after infection, the cultures were filtered through a cell strainer (pore size of 40 µm) to exclude the diatoms (empty frustules). The resulting sample, composed of *P. diadema* flagellates, was then filtered onto a Whatman Nuclepore Track-Etched Membrane (pore size of 0.8 µm) to exclude bacterial cells. *P. diadema* cells were scraped off the filter and used for the high-molecular-weight DNA extraction using the Qiagen MagAttract HMW DNA Kit (67563). DNA fragmentation was performed using g-TUBEs (Covaris) with a target size of 15 kb. Library preparation was performed using the SMRTbell Express TPK 2.0 and SMRTbell Enzyme Clean-up kit v1 (Pacific Biosciences). Sequencing was performed on a Sequel IIe PacBio machine. The ‘run design application’ was set to HiFi Reads with default settings. The resulting sequencing library comprised 739,524 reads with lengths up to 55,211 bases. Reads were filtered using ‘HiFiAdapterFilt’ to remove adapter-contaminated reads, resulting in 605,547 reads^62^. Bacterial contamination was removed using Kraken2^63^ with the ‘Standard plus’ RefSeq database that includes protozoa and fungi. The cleaned reads were assembled using HiFiasm v0.19.7^28^. BLASTn^60^ searches were carried against NCBI nt database^64^ to remove any bacterial and diatom contamination using BlobTools2^65^. The cleaned reads were reassembled resulting in an assembly of 5,155 contigs and assembly size of 171 Mb (N50 = 42.1 kb). BUSCO v6.0.0^66^ was used to assess the quality and completeness of the genome assembly relative to the stramenopile_odb10 database.

### *P. diadema* genome annotation

Annotation of the *P. diadema* genome assembly was performed using Funannotate v1.8.15 (https://github.com/nextgenusfs/funannotate). *P. diadema* transcripts (see below for details) were mapped to the genome using minimap2^67^ to generate homology-based gene predictions in the Program to Assemble Spliced Alignment^68^. Ab initio gene predictions were also performed using a combination of AUGUSTUS^69^, GeneMark-ES^70^, SNAP (https://github.com/KorfLab/SNAP), BUSCO^66^ and GlimmerHMM^71^. Transfer RNAs were predicted using tRNAscan-SE^72^. Evidence Modeler was used to consolidate the gene predictions and output consensus gene models^68^. The final gene set were functionally annotated using several databases (EGGNOG^73^, MEROPS^64^, InterProScan5^74^, CAZy^75^, Pfam^76^ and UniProt^77^). In addition, several of the putatively mispredicted genes were manually removed on the basis of the following criteria: (1) overrepresented orthologue copies, (2) lack of support from RNA-seq data, (3) lack of homology to other proteins and (4) lack of a predicted domain inside the protein. If all three of these conditions were met, the genes were excluded. The mitochondrial genome was assembled using MitoHifi^78^ and annotated using MFannot^79^ using the standard genetic code.

### Comparative genomics and phylogenomic tree construction

Pseudofungi proteomes were compiled from UniProt^77^, in addition to transcriptomes retrieved from Cho et al.^80^. Orthogroup inference was performed using OrthoFinder v2.5.5^33^ and upset plots were generated using the ComplexUpset^81^ package in RStudio v2023.12.1 + 402. GO enrichments were assessed by comparing the frequency of individual GO terms in *P. diadema*-specific genes to the whole proteome (that is, the background). Significance was assessed using permutation tests with test distributions produced by generating 100,000 randomized samples, equal in size to the test-set, by randomly selecting transcripts from the transcriptome without replacement. The highly enriched GO terms (*P* < 0.001) were summarized and visualized using REVIGO^82^.

For phylogenomic analyses, *P. diadema* proteins were searched against a set of 263 genes, previously used for eukaryote-wide phylogenetic analyses^18^ using BLASTp^60^. Resulting hits were filtered using an E-value threshold of 1 × 10^−20^ and a minimum query coverage of 50%. Individual alignments were produced using MAFFT^83^ and trimmed using trimAl^84^ (gap threshold of 0.6). Single gene trees were then constructed using IQ-TREE^85^ and manually inspected to remove paralogs and contaminants. The final cleaned gene-sets were filtered so that they contained a maximum of 60% missing positions and concatenated. The resulting concatenated alignment consisted of 241 genes spanning 79,399 amino acid positions from 45 taxa. A maximum likelihood phylogeny was generated from the concatenated alignment in IQ-TREE^85^ using the heterogeneous mixture LG + C40 + F + G4 model. Statistical support was assessed using 100 non-parametric bootstraps (NPBs) and 1,000 SH-like approximate likelihood ratio tests (SH-aLRT) support using the Q.yeast+F + I + R6 model as inferred by ModelFinder^86^. Phylogenomics statistics can be found in Supplementary Table 1, and alignments and phylogenies were deposited with Mendeley Data^58^.

### Host and parasite reference transcriptomics

*C. radiatus* cells were collected during both the light cycle (AM) and night cycle (PM) in three independent biological replicates. A total of 150 ml of dense *C. radiatus* cultures were filtered and washed on a cell strainer (pore size of 40 µm) and filtered on a membrane filter (pore size of 10 µm). The filter was directly immersed in 1.5 ml of Invitrogen TRIzol reagent and incubated at room temperature for 10 min followed by a 60 °C incubation for 3 min. The samples were centrifuged at 13,000*g* for 1 min to pellet the empty diatom frustules. *P. diadema* cells were harvested on day four from a 400 ml infected *C. radiatus* culture. The culture was filtered on a cell strainer (pore size of 40 µm) to exclude diatoms. The flow-through was then filtered (pore size of 10 µm) to exclude smaller diatom cells and broken frustules. The filtrate was centrifuged at 4,000*g* for 20 min, and the pellet was resuspended in 300 µl of Invitrogen TRIzol reagent. For both *C. radiatus* and *P. diadema*, RNA extraction and purification was performed using the Direct-zol RNA Miniprep kit (ZymoResearch). RNA concentration and quality was assessed with the Bioanalyzer RNA 6000 Nano assay kit and Qubit RNA Broad Range assay kit.

Illumina sequencing libraries were prepared using the Novogene NGS RNA Library Prep Set (PT042) and sequenced on the Illumina Novaseq 6000 platform. The resulting raw reads were trimmed using Cutadapt^87^ to remove adapter sequences. Kraken2^63^ was used to taxonomically classify the transcripts and remove bacterial contamination. The cleaned reads were assembled using rnaSPAdes^88^. Protein prediction were performed using TransDecoder (https://github.com/TransDecoder/TransDecoder). The proteomes underwent an additional round of bacterial contamination removal (and diatom contamination removal in the case of *P. diadema*) using Blob Toolkit^65^ and DIAMOND^89^ searches against the Swiss-Prot database^77^. The AM and PM *C. radiatus* datasets were merged for downstream analyses.

### Time-resolved dual bulk tramsciptomics

*C. radiatus* cultures were infected with 40 ml of *P. diadema* zoospores in three independent biological replicates. At 24 h after infection, 50 ml was subsampled from the infected culture and filtered on a membrane filter (pore size of 0.8 µm). In total, 1 ml of TRIzol reagent (Invitrogen) was added directly to the cells onto the filter and incubated at 60 °C for 2 min. Samples were centrifuged at 13,000*g* for 1 min to pellet diatom frustules and the downstream RNA extraction was carried out using the Direct-zol RNA Miniprep kit (ZymoResearch). The same process was repeated every 12 h until the end of the infection cycle at 96 h (for a total of six timepoints). The quality of the RNA was assessed with the Bioanalyzer RNA 6000 Nano assay kit and Qubit RNA Broad Range assay kit. Illumina sequencing libraries were prepared using poly-A selection Novogene NGS RNA Library Prep Set (PT042) and sequenced on the Illumina Novaseq 6000 platform.

### Differential expression and GO enrichment analysis

The raw Illumina paired-end sequencing reads were trimmed for adaptors and low-quality sequences using Cutadapt^87^. The expression of transcripts across samples (t1–t6) and replicates was quantified using Salmon^90^ by quasi-mapping the reads to combined reference of both host and parasite. The *Coscinodiscus* reference was derived from the reference transcriptomes generated in this study and the *Pirsonia* reference came from genes predicted from the genome. Trinity^91^ was used to perform cross-sample normalization (measured as transcripts per million) and to generate a transcript count matrix. DESeq2^35^, implemented in RStudio v2023.12.1 + 402, was used to detect DE transcripts and extract DE genes that were at least fourfold DE at a significance of ≤0.001 in any of the pairwise sample comparisons. DE genes were hierarchical clustered with hclust in R using the ward.D2 method. For GO enrichment analysis of the DE genes, we first annotated the *C. radiatus* and *P. diadema* proteomes with GO terms using Wei2GO^92^. This involved DIAMOND^89^ and HMMScan (http://hmmer.org/) searches against the UniProtKB^77^ and Pfam^76^ databases and using a weighting algorithm to calculate a score for the GO annotations. GO enrichment was assessed by comparing the frequency of individual GO terms in the DE genes to the whole proteome (that is the background). Significance was assessed using permutation tests with test distributions produced by generating 100,000 randomized samples, equal in size to the test-set, by randomly selecting transcripts from the transcriptome without replacement. The highly enriched GO terms (*P* < 0.001) were summarized and visualized using REVIGO^82^.

### Domain analysis and phylogeny of integrin-like proteins

To identify the protein domains present on *P. diadema* cell adhesion genes, profile HMM searches were performed using the Pfam^76^ database and HMMER^93^ (E-value threshold of 1 × 10^−5^ and domain E-value threshold of 1 × 10^−5^). The resulting domains were used to conduct HMM searches against our curated set of reference Pseudofungi proteomes (see above). Signal peptides and TMDs were predicted using DeepTMHMM v1^38^ and DeepLoc v2.1^39^. Next, we compiled a diverse reference dataset comprising proteomes from UniProt^77^. To taxonomically balance the dataset, we selected the best two proteomes per genus for eukaryotes (*n* = 141) on the basis of BUSCO^66^ completeness. For metazoans, fungi and embryophytes, more strict taxonomic criteria were set by selecting the best proteome per phyla for metazoans (*n* = 17), the best proteome per class for fungi (maximum two per phylum, *n* = 12) and the best proteome per order for embryophytes (maximum three per class, *n* = 11). For bacteria, the best proteome per species up to two per genus (*n* = 4,833) was selected, and for archaea, the best proteome per species up to two per genus (*n* = 310). For viruses, the best proteome per species up to one species was selected on the basis of protein count (*n* = 11,104). Each individual proteome was then clustered at 99% sequence identity using CD-HIT^94^.

The longest protein domain (PF13517: FG-GAP_3) on the integrin-like genes was used as a query for a BLASTp^60^ search against this curated reference dataset. The resulting hits were filtered at an E-value threshold of 1 × 10^−25^ and clustered at 97% sequence identity using CD-HIT^94^. The hits were aligned using MAFFT^83^ and trimmed with a gap threshold of 60% using trimAl^84^. The alignment was filtered to remove sequences that contained less than 50% of amino acid sites. ModelFinder^86^ was used to identify the best-fit evolutionary model according to the Bayesian information criterion, and a maximum likelihood phylogeny was constructed in IQ-TREE^85^ with support from 1,000 SH-aLRT and 1,000 ultrafast bootstraps (UFBs). Eukaryotic OTUs forming clades with single species branching within prokaryotes and vice versa for prokaryotic sequences were excluded. Tree editing and visualization were done in iTOL v6^95^. Alignments and phylogenies were deposited in Mendeley Data^58^.

### U-ExM

*C. granii* cultures (20 ml) were infected with *P. diadema* zoospores (2 ml), cells were sampled across the 4-day infection cycle. Cells were prepared using either cryo-fixation or chemical fixation. Cryo-fixation and cryo-substitution were performed following established diatom U-ExM protocols^40^. In brief, cells were mounted on 12-mm poly-l-lysine-coated coverslips, plunge-frozen in liquid ethane using a manual plunge freezer (RHOST LLC) and freeze-substituted overnight in frozen acetone containing 0.5% (v/v) formaldehyde and 0.025% (v/v) glutaraldehyde under dry ice, followed by gradual warming over 24 h and rehydration through a graded ddH_2_O series to PBS. For visualization of actin using HAK-actin (Spirochrome, SC019)^41^, late-stage infections (day 3) were chemically fixed in 4% (v/v) paraformaldehyde and 0.025% (v/v) glutaraldehyde for 1 h at 4 °C, mounted on poly-L-lysine-coated coverslips, permeabilized in PBS with 0.1% (v/v) Tween 20. HAK-actin (1:500) was then applied to the fixed and permeabilized samples for 45 min. Samples were subsequently post-fixed and processed for U-ExM as previously described^49^. Immunolabelling was performed in PBS containing 4% (w/v) BSA and 0.05% (v/v) Tween 20 using mouse anti-α-tubulin (12G10; DHSB; AB_1157911; 1:1,000), rabbit anti-HA antibody (HA Tag Recombinant Rabbit Monoclonal Antibody RM305; Invitrogen/Thermo Fisher Scientific, MA5-27915; Research Resource Identifier, AB_2744968; clone RM305; 1:566), followed by Alexa Fluor 488 and Alexa Fluor 555 conjugated secondary antibodies (Thermo Fisher Scientific; 1:1,000). Samples were washed in PBS containing 0.1% Tween 20, counterstained with NHS-ester Alexa Fluor 647 (1 µg ml^−^^1^) and DAPI (5 µg ml^−^^1^), reexpanded in ddH_2_O and imaged on a Nikon CSU-W1 SORA spinning-disk confocal microscope using 40× or 60× water-immersion objectives. Image analysis was performed in FIJI (ImageJ v1.54)^59^. There are two scale bars associated with each image of an expanded cell. The ‘expanded’ scale bar is the scale bar produced by the confocal during image acquisition. The ‘biological’ scale bar estimates the actual size of the objects in the image given the known expansion factor of the gel (expansion factor = expanded gel diameter/initial coverslip diameter). The formula ‘biological scale bar = expanded scale bar/expansion factor’ can be used to convert between the two, where the expansion factor is unique to each gel.

### Reporting summary

Further information on research design is available in the Nature Portfolio Reporting Summary linked to this article.

## Supplementary information


Reporting summary
Supplementary Table 1Pirsonia OTUs in Tara Oceans expeditions. **a**, *Pirsonia* OTU sequences, total reads and presence in Tara Oceans samples. **b**, Tara Oceans samples positive for *Pirsonia* and the corresponding geographical coordinates.
Supplementary Table 2Phylogenomics tree statistics. **a**, Species included in the phylogenomic tree and their representation (%) across the 242 genes. **b**, Genes used for phylogenomics and percentage of species represented per gene.
Supplementary Table 3Orthogroup composition across reference stramenopile proteomes inferred using OrthoFinder. **a**, List of orthogroups and the corresponding proteins identified from the reference stramenopile proteomes (available on Mendeley Data^58^). **b**, The number of proteins per orthogroup for each species.
Supplementary Table 4*P. diadema* transcriptomics. **a**, The FPKM-normalized gene expression matrix. **b**, The gene expression clusters and the list of genes assigned to each of the four clusters. **c**, GO terms enriched in each expression cluster. **d**, The raw read count matrix used for differential expression analysis
Supplementary Table 5*Pirsonia* integrin proteins and localization predictions. Protein identifiers, sequences, lengths, genomic scaffolds, intron numbers, DeepLoc2.1-based localization, signal and membrane-type predictions and the number of predicted transmembrane regions.
Supplementary Table 6*C. radiatus* transcriptomics. **a**, The FPKM-normalized gene expression matrix. **b**, The list of DE genes; corresponding protein sequences are available on Mendeley Data^58^. **c**, The GO terms enriched among the DE genes.
Supplementary Video 1HCS time-lapse of *P. diadema* infection of *C. granii*. Images were acquired every 6 h over a total duration of 96 h at 18 °C. Brightfield and Cy5 channels (chlorophyll autofluorescence) are shown. Scale bar, 200 µm.


## Source data


Source Data Extended Data Fig./Table 1Source data for Fig. 2c showing accessions of publicly available datasets used for the upset plot and for Fig. 2h showing protein length distributions in species-specific and non-species-specific orthogroups across *P. diadema* and three oomycete species. The file includes the plotted orthogroup-level protein length values, sample sizes and statistical test results from two-sided Welch two-sample *t*-tests.
Source Data Extended Data Fig./Table 2Source data for Fig. 5a–e showing cytoskeletal perturbation assay measurements. The file includes biological replicate well-level values and summary statistics for diatom survival, infection dynamics, normalized zoospore counts and timed cytochalasin D addition assays, as well as individual zoospore track measurements and Wilcoxon rank-sum test results for motility analysis.


## Data Availability

Raw PacBio genome sequencing reads for *P. diadema* are available in the NCBI Sequence Read Archive under accession number SRR34520686. All transcriptomic raw reads are available under BioProject accessions PRJNA1291136, PRJNA1290282 and PRJNA1292109. Gene predictions, annotations, predicted proteins, phylogenomic gene alignments and trees and all Python and R analysis scripts are available on Mendeley Data^58^. *C. granii* and *P. diadema* cultures are available from the authors upon request. Source data are provided with this paper.
