# Supplementary material for: Molecular and cellular insights into the parasitic infection of bloom-forming marine diatoms by Pirsonia diadema
Source: Nat Microbiol. 2026 Jul 21;11(9):2478–92. doi: 10.1038/s41564-026-02421-4 (PMC13524835; doi:10.1038/s41564-026-02421-4)
Supplement: Supplementary file 1 [file 41564_2026_2421_MOESM1_ESM.pdf]

nature portfolio

Corresponding author(s): Varsha Mathur

Last updated by author(s): Feb 23, 2026

Reporting Summary

Nature Portfolio wishes to improve the reproducibility of the work that we publish. This form provides structure for consistency and transparency in reporting. For further information on Nature Portfolio policies, see our Editorial Policies and the Editorial Policy Checklist.

Statistics

For all statistical analyses, confirm that the following items are present in the figure legend, table legend, main text, or Methods section.

n/a	Confirmed	
☐	☒	The exact sample size (*n*) for each experimental group/condition, given as a discrete number and unit of measurement
☐	☒	A statement on whether measurements were taken from distinct samples or whether the same sample was measured repeatedly
☐	☒	The statistical test(s) used AND whether they are one- or two-sided<br>*Only common tests should be described solely by name; describe more complex techniques in the Methods section.*
☒	☐	A description of all covariates tested
☐	☒	A description of any assumptions or corrections, such as tests of normality and adjustment for multiple comparisons
☐	☒	A full description of the statistical parameters including central tendency (e.g. means) or other basic estimates (e.g. regression coefficient) AND variation (e.g. standard deviation) or associated estimates of uncertainty (e.g. confidence intervals)
☐	☒	For null hypothesis testing, the test statistic (e.g. *F*, *t*, *r*) with confidence intervals, effect sizes, degrees of freedom and *P* value noted<br>*Give P values as exact values whenever suitable.*
☒	☐	For Bayesian analysis, information on the choice of priors and Markov chain Monte Carlo settings
☐	☒	For hierarchical and complex designs, identification of the appropriate level for tests and full reporting of outcomes
☒	☐	Estimates of effect sizes (e.g. Cohen's *d*, Pearson's *r*), indicating how they were calculated

Our web collection on statistics for biologists contains articles on many of the points above.

Software and code

Policy information about availability of computer code

Data collection

Microscopy data were collected using multiple imaging platforms. Confocal imaging was performed on a Zeiss LSM 780 confocal microscope using ZEN software and on a Nikon CSU-W1 SORA spinning-disk system using NIS-Elements software. High-content time-series imaging for life-cycle assays was conducted on an ImageXpress Pico Automated Cell Imaging System (Molecular Devices) using CellReporterXpress software for image stitching. Imaging for cytoskeletal drug assays was performed on a Zeiss Cell Discoverer 7 microscope or Zeiss LSM 800 confocal microscope using ZEN software. No custom code was used for data collection beyond manufacturer-provided software.

Data analysis

Microscopy data were analyzed using Fiji (ImageJ v1.54) and the Labkit plugin for image segmentation. Manufacturer-provided imaging software (ZEISS ZEN and CellReporterXpress) was used for image acquisition and stitching. Genomic and transcriptomic analyses were performed using open-source software, including Kraken2, BlobTools2, HiFiasm v0.19.7, Funannotate v1.8.15, BUSCO v6.0.0, Salmon v1.10.1, DESeq2 v1.41.12, ggplot2 v3.5.2, R v2023.12.1+402, and Python v3.13.3. Phylogenetic and comparative genomics analyses used AliView v1.28, IQ-TREE v2, MAFFT v7, OrthoFinder v2.5.5, CD-HIT v4.8.1, HMMER v3, DeepTMHMM v1, DeepLoc v2.1, trimAl v1.2, iTOL v4, and REVIGO. All custom code has been made publicly available via the Mendeley Data repository.

For manuscripts utilizing custom algorithms or software that are central to the research but not yet described in published literature, software must be made available to editors and reviewers. We strongly encourage code deposition in a community repository (e.g. GitHub). See the Nature Portfolio guidelines for submitting code & software for further information.

Data

Policy information about availability of data

All manuscripts must include a data availability statement. This statement should provide the following information, where applicable:

- Accession codes, unique identifiers, or web links for publicly available datasets
- A description of any restrictions on data availability
- For clinical datasets or third party data, please ensure that the statement adheres to our policy

Raw PacBio genome sequencing reads for P. diadema are available in the NCBI Sequence Read Archive under accession SRR34520686. All transcriptomic raw reads are available under BioProject accessions PRJNA1291136, PRJNA1290282, and PRJNA1292109. Gene predictions, genome annotations, predicted protein sequences, phylogenomic gene alignments and trees, and all Python and R analysis scripts are available on Mendeley Data (doi: 10.17632/xc89mvhxpg). Host and parasite cultures are available from the authors upon reasonable request.

Research involving human participants, their data, or biological material

Policy information about studies with human participants or human data. See also policy information about sex, gender (identity/presentation), and sexual orientation and race, ethnicity and racism.

Reporting on sex and gender	This study did not involve human participants, human data, or human biological material.
Reporting on race, ethnicity, or other socially relevant groupings	This study did not involve human participants, human data, or human biological material.
Population characteristics	This study did not involve human participants, human data, or human biological material.
Recruitment	This study did not involve human participants, human data, or human biological material.
Ethics oversight	This study did not involve human participants, human data, or human biological material.

Note that full information on the approval of the study protocol must also be provided in the manuscript.

Field-specific reporting

Please select the one below that is the best fit for your research. If you are not sure, read the appropriate sections before making your selection.

☐ Life sciences ☐ Behavioural & social sciences ☒ Ecological, evolutionary & environmental sciences

For a reference copy of the document with all sections, see nature.com/documents/nr-reporting-summary-flat.pdf

Ecological, evolutionary & environmental sciences study design

All studies must disclose on these points even when the disclosure is negative.

Study description	This study examines interactions between the diatom Coscinodiscus and its parasite Pirsonia diadema using microscopy, genomics, and transcriptomics. Quantitative analyses focused on infection dynamics under cytoskeletal drug treatments. Time-series imaging was performed every 6 h for up to 96 h. Experimental units consisted of individual wells containing host and parasite cells, with repeated measurements across multiple time points. Transcriptomes of host and parasite were sampled across a time series and during the host day/night cycle. Replication included three biological replicates for infection experiments and multiple technical replicates for sequencing.
Research sample	Clonal populations of the host diatoms Coscinodiscus radiatus and Coscinodiscus granii, and the parasite Pirsonia diadema, were used. All cultures were derived from single cells isolated from plankton tows.
Sampling strategy	Sample sizes for microscopy and infection assays were determined based on practical considerations. Each experimental condition was performed in triplicate wells, providing biological replication. Time-series imaging followed individual cells within the same wells across multiple time points, resulting in repeated measures. No formal sample size calculation was performed for transcriptomic experiments; culture densities and replication were consistent with established protocols in the field.
Data collection	Data collection was performed by the authors. Microscopy data were acquired using scanning electron microscopy and confocal microscopy on fixed and live infected and uninfected diatom samples. Time-lapse high-content imaging was performed using automated imaging systems (ImageXpress Pico and Zeiss Cell Discoverer 7). For genomic data, DNA and RNA were extracted from host and parasite cultures using commercial kits (Qiagen MagAttract HMW DNA Kit and Direct-zol RNA Miniprep). Library preparation and sequencing were performed by sequencing facility staff using PacBio Sequel IIe and Illumina NovaSeq platforms.
Timing and spatial scale	Coscinodiscus species were isolated from plankton tows in the Western English Channel in January 2022 and December 2024. Pirsonia diadema was isolated from plankton tows in Yongho Bay in November 2022. Laboratory infection experiments were

	conducted under controlled conditions, with temporal sampling spanning the infection cycle over 96 h and imaging performed every 6 h. Genome and transcriptome sequencing were conducted on samples harvested at defined infection time points between 24 and 96 h post-infection. Environmental survey data from the Tara Oceans Expedition were used to extend the spatial scale to a global oceanic context.
Data exclusions	Contaminant sequences were excluded during data processing. For genomic and transcriptomic datasets, bacterial reads were identified and removed using Kraken2 and BlobTools. Adapter-contaminated and low-quality reads were filtered using HiFiAdapterFilt and Cutadapt. For phylogenomic analyses, paralogous sequences, contaminants, and low-quality sequences were manually curated and excluded based on sequence similarity and alignment quality. During image analysis, outliers in fluorescence intensity and cell area were excluded using an interquartile range filter.
Reproducibility	Experimental reproducibility was addressed through biological replication at each time point in the infection time course, with three independent cultures sampled across six time points. RNA-seq data processing, differential expression, and gene ontology analyses were performed using established, version-controlled open-source software and standardized workflows. Genome and transcriptome assemblies were generated using independent runs of HiFiasm and rnaSPAdes, and assembly quality metrics were assessed. Image-based phenotyping used automated segmentation and quantification pipelines.
Randomization	Randomization was not applicable to this study, as the experimental design involved time-resolved sampling of infected cultures across a defined infection cycle rather than random allocation to treatment groups. All cultures were maintained under identical environmental conditions, and replicates were processed in parallel to minimize technical variability and batch effects.
Blinding	Blinding was not applicable to this study, as samples or organisms were not allocated to experimental groups.

Did the study involve field work? ☒ Yes ☐ No

Field work, collection and transport

Field conditions	Surface ocean plankton-net tows
Location	Coscinodiscus radiatus and Coscinodiscus granii were isolated from plankton tows at Station L4 in the Western English Channel (Plymouth, UK). Pirsonia diadema was isolated from plankton tows in Yongho Bay (Busan, Republic of Korea).
Access & import/export	Plankton samples were collected in the United Kingdom and the Republic of Korea for non-commercial academic research. No special permits were required at the time of collection, and sampling was conducted in accordance with national legislation and institutional guidelines. The Pirsonia diadema isolate from the Republic of Korea was obtained through an academic collaboration with Prof. Sunju Kim at Pukyong National University.
Disturbance	No disturbance to the environment occurred during plankton collection.

Reporting for specific materials, systems and methods

We require information from authors about some types of materials, experimental systems and methods used in many studies. Here, indicate whether each material, system or method listed is relevant to your study. If you are not sure if a list item applies to your research, read the appropriate section before selecting a response.

Materials & experimental systems

n/a	Involved in the study	
☐	☒	Antibodies
☒	☐	Eukaryotic cell lines
☒	☐	Palaeontology and archaeology
☒	☐	Animals and other organisms
☒	☐	Clinical data
☒	☐	Dual use research of concern
☒	☐	Plants

Methods

n/a	Involved in the study	
☒	☐	ChIP-seq
☒	☐	Flow cytometry
☒	☐	MRI-based neuroimaging

Antibodies

Antibodies used	Primary antibodies: mouse anti-α-tubulin antibody (12G10; Developmental Studies Hybridoma Bank, DSHB; RRID: AB_1157911; clone 12G10; 1:1000); rabbit anti-HA antibody (HA Tag Recombinant Rabbit Monoclonal Antibody RM305; Invitrogen/Thermo Fisher Scientific; Cat. No. MA5-27915; RRID: AB_2744968; clone RM305; 1:566). Secondary antibodies: anti-mouse IgG Alexa Fluor 555-conjugated secondary antibody (Thermo Fisher Scientific; RRID: AB_2633276; 1:1000); anti-rabbit IgG Alexa Fluor 488-conjugated secondary antibody (Thermo Fisher Scientific; RRID: AB_2576217 and/or RRID: AB_143157; 1:1000).
Validation	Primary antibody validation links: mouse anti-α-tubulin 12G10, DSHB, RRID: AB_1157911, https://scicrunch.org/resolver/

Validation

AB_1157911; rabbit anti-HA RM305, Invitrogen/Thermo Fisher Scientific, Cat. No. MA5-27915, RRID: AB_2744968, https://www.thermofisher.com/antibody/product/HA-Tag-Antibody-clone-RM305-Recombinant-Monoclonal/MA5-27915.

Plants

Seed stocks

This study did not involve plants.

Novel plant genotypes

This study did not involve plants.

Authentication

This study did not involve plants.
